# Regulation of the RNAPII Pool Is Integral to the DNA Damage Response

**DOI:** 10.1016/j.cell.2020.02.009

**Published:** 2020-03-19

**Authors:** Ana Tufegdžić Vidaković, Richard Mitter, Gavin P. Kelly, Michelle Neumann, Michelle Harreman, Marta Rodríguez-Martínez, Anna Herlihy, Juston C. Weems, Stefan Boeing, Vesela Encheva, Liam Gaul, Laura Milligan, David Tollervey, Ronald C. Conaway, Joan W. Conaway, Ambrosius P. Snijders, Aengus Stewart, Jesper Q. Svejstrup

**Affiliations:** 1 Mechanisms of Transcription Laboratory, The Francis Crick Institute, 1 Midland Road, London NW1 1AT, UK; 2 Bioinformatics and Biostatistics, The Francis Crick Institute, 1 Midland Road, London NW1 1AT, UK; 3 Protein Analysis and Proteomics Laboratory, The Francis Crick Institute, 1 Midland Road, London NW1 1AT, UK; 4 Stowers Institute for Medical Research, Kansas City, MO 64110, USA; 5 Department of Biochemistry and Molecular Biology, University of Kansas Medical Center, Kansas City, KS 66160, USA; 6 Wellcome Trust Centre for Cell Biology, University of Edinburgh, Edinburgh, Scotland

**Keywords:** RNA polymerase II, ubiquitylation, DNA damage, transcription, UV irradiation, ubiquitin

## Abstract

In response to transcription-blocking DNA damage, cells orchestrate a multi-pronged reaction, involving transcription-coupled DNA repair, degradation of RNA polymerase II (RNAPII), and genome-wide transcription shutdown. Here, we provide insight into how these responses are connected by the finding that ubiquitylation of RNAPII itself, at a single lysine (RPB1 K_1268_), is the focal point for DNA-damage-response coordination. K_1268_ ubiquitylation affects DNA repair and signals RNAPII degradation, essential for surviving genotoxic insult. RNAPII degradation results in a shutdown of transcriptional initiation, in the absence of which cells display dramatic transcriptome alterations. Additionally, regulation of RNAPII stability is central to transcription recovery—persistent RNAPII depletion underlies the failure of this process in Cockayne syndrome B cells. These data expose regulation of global RNAPII levels as integral to the cellular DNA-damage response and open the intriguing possibility that RNAPII pool size generally affects cell-specific transcription programs in genome instability disorders and even normal cells.

## Highlights

• Specific RPB1 K1268 ubiquitylation targets RNAPII for UV-induced proteolysis
• RPB1 K1268 ubiquitylation is required for surviving DNA damage
• Control of the RNAPII pool via degradation regulates the transcriptome after UV
• Lack of transcription recovery in Cockayne syndrome is caused by unstable RNAPII

## Graphical Abstract

**Figure:**
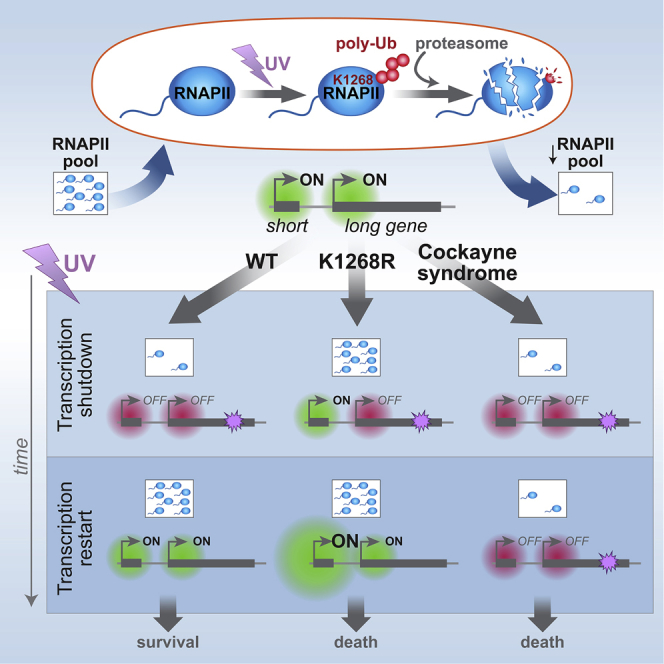

## Teaser

Control of the pool of available RNA polymerase II shapes how cells respond to UV stress and the efficacy of the resulting damage response.

## Introduction

DNA damage, such as the bulky lesions generated by UV irradiation, not only elicit DNA repair but also dramatically affect transcriptional output. Upon UV exposure, a global “shutdown” of transcription occurs—an immediate inhibition of transcription elongation (Lavigne et al., 2017, Williamson et al., 2017), followed by inhibition of transcription initiation, even on undamaged genes (Gyenis et al., 2014, Rockx et al., 2000, Williamson et al., 2017). The purpose and mechanism of these phenomena have remained obscure.

For cells to survive UV irradiation, they must ultimately recover transcription activity, a process that fails in individuals suffering from Cockayne syndrome (Mayne and Lehmann, 1982). Several factors have been implicated in transcription recovery upon DNA damage, most notably Cockayne syndrome B protein (CSB) itself (Proietti-De-Santis et al., 2006). Other transcription factors, chromatin remodelers, and non-coding RNAs play a role in re-establishing the active transcriptional state as well (Adam et al., 2013, Dinant et al., 2013, Epanchintsev et al., 2017, Mourgues et al., 2013, Oksenych et al., 2013, Williamson et al., 2017), yet the mechanisms remain elusive.

In parallel to transcription changes, DNA damage triggers two other processes centered around lesion-stalled RNAPII: transcription-coupled nucleotide excision repair (TC-NER) and RNAPII ubiquitylation and degradation (for review, see Gregersen and Svejstrup, 2018). The former process preferentially repairs DNA lesions in the transcribed strand of active genes (Gregersen and Svejstrup, 2018, Mellon et al., 1987, Vermeulen and Fousteri, 2013), and the latter removes damage-stalled RNAPII from chromatin, presumably in situations where TC-NER is unsuccessful (Wilson et al., 2013a). Nonetheless, the importance of this pathway in human cells is not fully understood, because it has not been possible to specifically modulate it and observe the consequences.

Here, we uncover a single ubiquitylation site in the largest, catalytic subunit of human RNAPII, RPB1 lysine 1268 (K_1268_) and show that it is required for RNAPII poly-ubiquitylation and degradation and important for surviving UV irradiation. Surprisingly, K_1268_ubiquitylation profoundly affects the global transcriptional response to UV: regulation of global RNAPII levels via K_1268_ ubiquitylation is central to the initial UV-induced transcription shutdown and also for later transcription recovery. Indeed, CSB-deficient cells fail to restart transcription largely due to a persistent decrease in the overall RNAPII pool.

## Results

### K_1268_ Is Required for UV-Induced RPB1 Poly-ubiquitylation and Degradation

**Figure 1:**
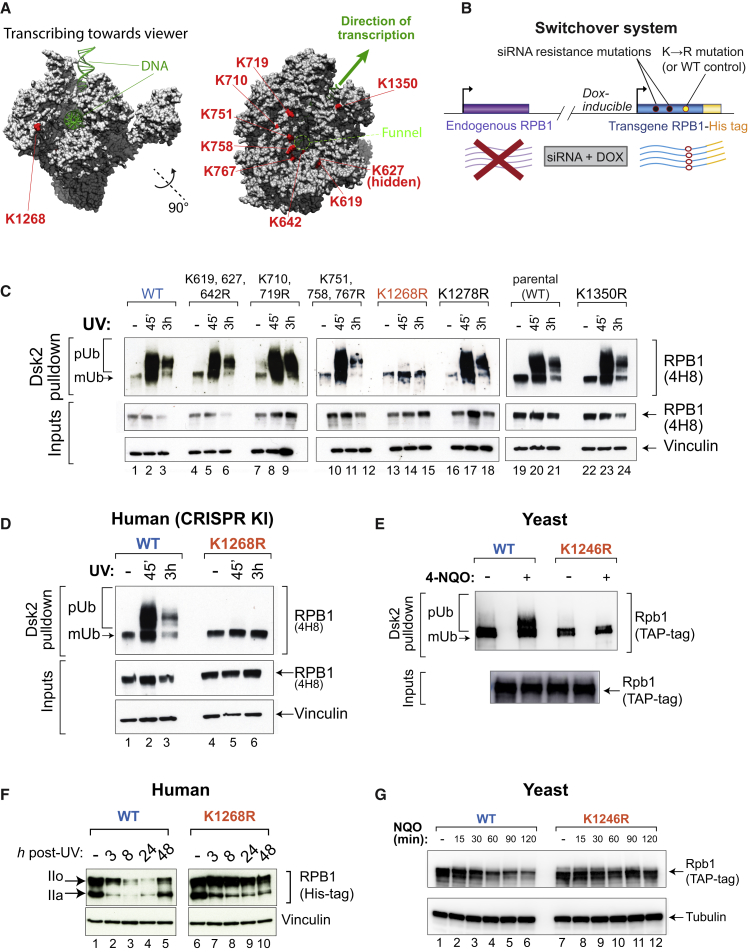
RPB1 K_1268_ Is Important for UV-Induced Poly-ubiquitylation and Degradation (A) UV-induced RPB1 ubiquitylation sites (red) on the mammalian RNAPII structure (Bernecky et al., 2016). (B) Schematic of the RPB1 switchover system. (C) Dsk2 pulldown-western blot analysis of cells expressing RPB1 with different K → R mutations, before and after UV irradiation (20 J/m^2^). *K*_*1350*_*R* is a CRISPR KI, matched with its own control. (D) As in (C) but in *K*_*1268*_*R* CRISPR KI cells. (E) As in (C) and (D), but in yeast, before and after 4-NQO treatment (10 μg/mL). (F) Western blot analysis of UV-induced RPB1 degradation after 20 J/m^2^ UV irradiation. Switchover cells were used as outlined in Figure S1A. Total RPB1 is detected with the anti-His tag antibody. Vinculin is the loading control. (G) Western blot analysis of yeast TAP-Rpb1 degradation after treatment with 10 μg/mL of 4-NQO. Tubulin is the loading control. See also Figure S1 and Table S1.

A small subset of RNAPII molecules that arrest at DNA lesions is ubiquitylated on their largest subunit, RPB1 (POLR2A), yet the functionally important sites of ubiquitylation are unknown. To chart these sites, we overlapped our own RPB1 ubiquitylation profiling data (Boeing et al., 2016), with other similar studies (Elia et al., 2015, Povlsen et al., 2012), which yielded 10 high-confidence sites (Figure 1A; Table S1).

**Figure S1:**
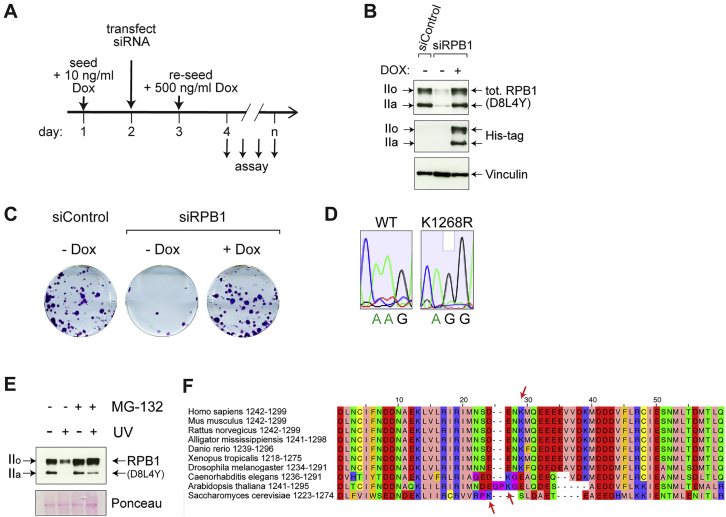
K_1268_ Is a Major or Sole Signal for UV-Induced RPB1 Poly-ubiquitylation and Degradation, Related to Figure 1 (A) Experimental setup: siRNA and doxycycline treatments in K → R switchover model system cell lines. (B) Western blot showing the efficiency of the switchover model system (in this example WT switchover control – K → K), two days after transfection (day 4, see A), in whole cell extracts. Total (D8L4Y) and transgenic (His-tagged) RPB1 were detected. Vinculin is used as a loading control. (C) Colony formation assay showing the efficiency of the switchover system in supporting cell survival (in this example WT switchover control – K → K is shown). (D) Sanger sequencing traces of the genomic DNA region encoding RPB1 K_1268_ (AAG) and the corresponding K → R mutation (AGG). Parental cells (WT) and a CRISPR knock-in clone E2 are shown. (E) Western blot showing levels of RPB1 (D8L4Y antibody) on chromatin in WT cells, before and after proteasome inhibition (MG-132) and UV treatments. Cells were pre-treated with 5 μM MG-132 for 3 h, then treated with 20 J/m^2^ UV. Extracts were prepared 3 hours after UV. (F) Sequence alignment of the RPB1 unstructured loop region across representative eukaryote species. The presence of lysine (K) corresponding to human K_1268_ is marked with arrows.

To investigate their functional importance, we used a “switchover” model system in which endogenous RPB1 is replaced with a transgenic version carrying lysine to arginine (K → R) mutation to prevent ubiquitylation. Switchover is achieved with small interfering RNAs (siRNAs) against the endogenous RPB1 transcript and doxycycline (Dox) addition to express a stably integrated, siRNA-resistant *RPB1* transgene encoding 6xHis-tagged RPB1 (Figures 1B, S1A, and S1B). Near-complete switchover was achieved, with expression at near-endogenous levels (Figure S1B), and the wild-type (WT) *RPB1* transgene supported cell survival (Figure S1C). Cell lines expressing RPB1 with K→R mutation at one or more ubiquitylation sites (Figure 1A) were generated. Ubiquitylated proteins from switchover cell extracts were isolated using Dsk2 pulldown (Anindya et al., 2007, Tufegdzic Vidakovic et al., 2019) and RPB1 ubiquitylation was analyzed by western blotting. Strikingly, a single K → R substitution, at K_1268_, almost completely abolished UV-induced RPB1 poly-ubiquitylation while other K → R substitutions had little or no effect (Figure 1C). Cell lines expressing RPB1 K_1268_R from the endogenous *POLR2A* locus were generated using CRISPR knockin (KI) technology (Figure S1D), which dramatically affected UV-induced RPB1 poly-ubiquitylation as well (Figure 1D).

Induction of RPB1 poly-ubiquitylation in response to transcription stress is conserved from yeast to humans (Wilson et al., 2013a). Indeed, mutation of the site analogous to human RPB1 K_1268_ (i.e., Rpb1 K_1246_) (Milligan et al., 2017), affected yeast Rpb1 ubiquitylation in response to the UV-mimicking agent 4-nitroquinoline N-oxide (4-NQO) (Figure 1E).

Analysis of RPB1 protein levels at different time points after UV exposure showed proteasome-mediated RPB1 degradation in WT cells, which was clearly visible from 3 h onward after treatment (Figures 1F, left, and S1E). In stark contrast, *K*_*1268*_*R* cells were deficient for RPB1 degradation (Figure 1F, right panel). K_1246_ was required for Rpb1 degradation in yeast as well (Figure 1G), showing that the process is conserved. Interestingly, K_1268_/K_1246_ is located in an unstructured loop protruding from the surface of RPB1, apparently a conserved feature (Bernecky et al., 2016, Cramer et al., 2001, Gnatt et al., 2001). While the primary loop sequence varies across species, the presence of a lysine somewhere in the loop is preserved (Figure S1F). These results show that human RPB1 K_1268_ represents a single, conserved receptor site for DNA damage-induced poly-ubiquitylation, necessary for RPB1 degradation after DNA damage.

### K_1268_ Ubiquitylation Is Required for Cell Survival upon DNA Damage and Affects the Rate of DNA Repair

Various roles for RPB1 poly-ubiquitylation and degradation in the cellular response to DNA damage have been proposed (Bregman et al., 1996, Wilson et al., 2013a, Woudstra et al., 2002), but the *K*_*1268*_*R* cells now provided us with the opportunity to specifically modulate the process and study the consequences.

**Figure 2:**
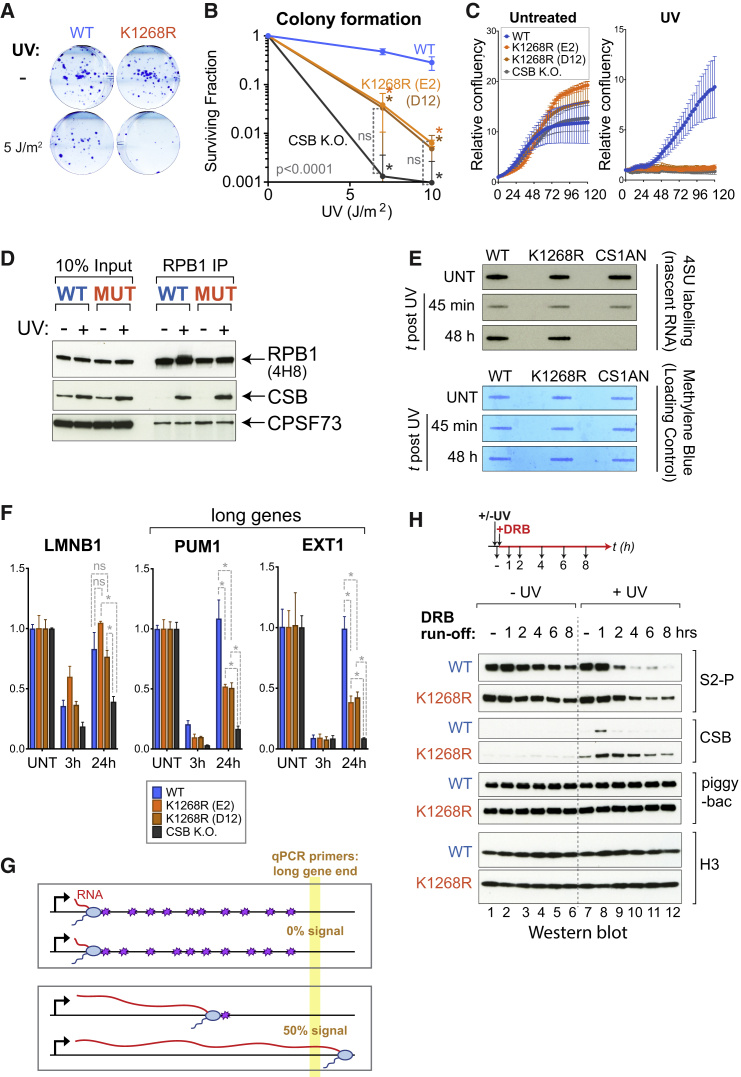
K_1268_ Ubiquitylation Is Required for Cell Survival upon DNA Damage and Affects Repair Kinetics (A) Representative images of colony formation assays before and after UV irradiation (5 J/m^2^) (switchover system). (B) Quantification of colony formation assays (n = 3) as in (A), but using *K*_*1268*_*R* CRISPR KI cells and CSB KO control cells. Data are presented on a log_10_ scale, as average surviving fractions ± SD. Asterisks indicate significance of differences (comparison versus WT cells) (p < 0.05, Tukey two-way ANOVA). ns, not significant (comparison versus CSB KO cells). (C) Growth assays before (left panel) and after UV irradiation (20 J/m^2^) (right panel). Cell confluency was monitored every 3 h using Incucyte and the data were normalized to t = 0 for each well. Data are represented at each 3 h time point as average relative confluency of 3 biological replicates ± SD. (D) Immunoprecipitation (IP) of RPB1 from chromatin, followed by western blot for RPB1, CSB and CPSF73 (control). Cells were UV-irradiated (20 J/m^2^, or not) and collected 45 min later for IP. (E) 4SU-slotblot showing global nascent RNA production before and after UV irradiation (10 J/m^2^). Cells were pulse-labeled with 4SU 15 min prior to collection. Methylene blue staining is the loading control. (F) RT-qPCR measuring nascent transcription before or after UV-irradiated (20 J/m^2^) at *LMNB1* (60 kb), *EXT1* (317 kb), and *PUM1* (134 kb), using primers at their 3′ ends. Data are represented as mean ± SD, normalized to the mature GAPDH transcript, and to untreated conditions. Statistically significant differences (three biological replicates) are indicated with asterisks (p < 0.05, multiple t tests, Holm-Sidak correction). (G) Schematic illustrating the relationship between DNA damage burden (purple stars) and nascent transcription on a long gene. Restart is only detected when all lesions have been removed; 50% restart indicates that all lesions have been removed from 50% of genes in the cell population. (H) Experimental approach (top) and western blot analysis (bottom), with DRB added immediately after UV irradiation, and samples collected at the indicated time points. The abundance of S2-phosphorylated (S2_P_, 3E10) RPB1, as well as CSB and histone H3 (control) in chromatin is shown. Piggybac, product of transposon insertion into the *CSB* locus, was used as loading/specificity control. See also Figure S2.

**Figure S2:**
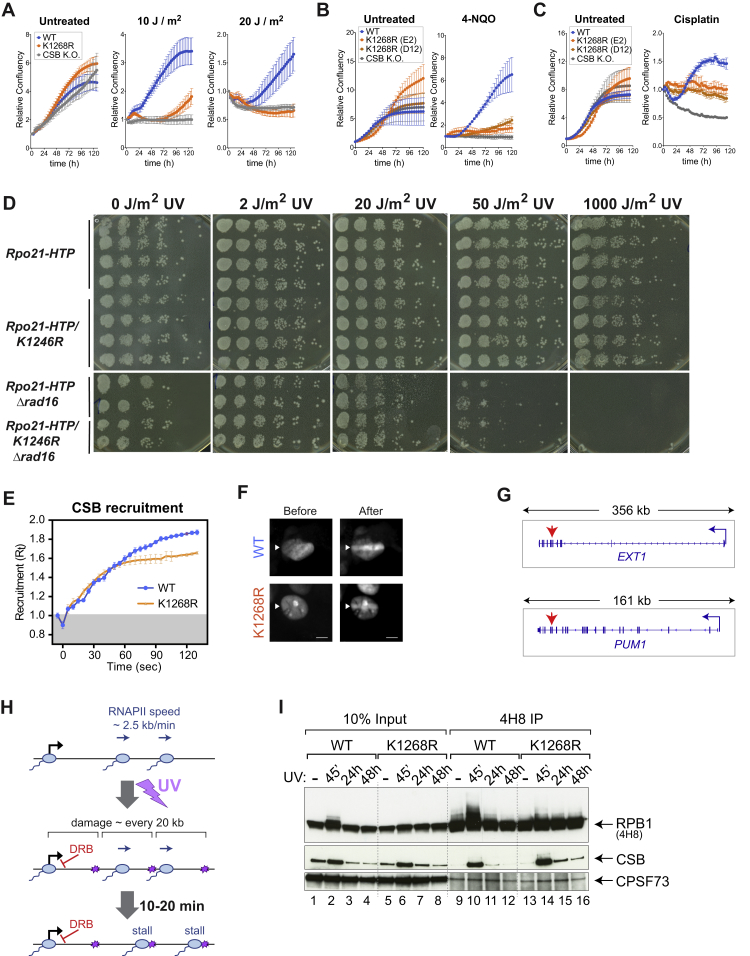
K_1268_ Ubiquitylation Is Required for Cell Survival upon DNA Damage but Not for TC-NER, Related to Figure 2 (A) Growth assays before and after UV irradiation (10 J/m^2^ and 20 J/m^2^), in WT and *K*_*1268*_*R* switchover model systems and CSB knock-out cells. Cell growth (confluency) was monitored every 3 h using Incucyte and the data were normalized to t = 0 for each well. Data are represented at each 3h time point as average relative confluency of 3 biological replicates ± SD. Please note that normalization to t = 0 results in technical variability between samples, such as the impression that *K*_*1268*_*R* cells grow better than WT cells in untreated condition, however this is not significant or reproducible. (B and C) Growth assays before and after the treatment with 4-NQO (0.5 μM for 1 h) (B) or cisplatin (90 μM for 1 h) (C), in WT and *K*_*1268*_*R* CRISPR knock-in and CSB knock-out cells. Cell growth (confluency) was monitored every 4 h (B) or every 3 h (C) using Incucyte and the data were normalized to t = 0 for each well. Data are represented at each 3h/4h time point as average relative confluency of 3 biological replicates ± SD (D) Sensitivity of yeast cells with the genotype shown on the left, to the levels of UV irradiation shown above. (E) Recruitment of GFP-tagged CSB in either parental HEK293T cells (WT, Blue) or in cells carrying the K_1268_R RPB1 mutation (K1268R, Orange). Micro-irradiation was initiated at time t = 0, and cells were imaged every second, with intensity values binned over 5 s intervals. Graphs show mean ± SEM, n = 18 cells (6 cells from each of 3 independent experiments). (F) Representative images of either WT or *K*_*12*__*68*_*R* cells before and after being subjected to micro-irradiation; white triangles indicate regions of micro-irradiation, scale bars, 8 μm. (G) Gene browser snapshots showing the location of primers (red arrows) used for measuring transcription restart on two long genes, *EXT1* and *PUM1*. (H) A sketch depicting the time frame within which all RNAPII will stall at DNA damages, upon UV irradiation of 20 J/m^2^. Addition of DRB in the DRB run-off experiment, blocking the new release of RNAPII into elongation, is indicated in red. (I) Immunoprecipitation (IP) of RPB1 from chromatin fractions followed by western blot for RPB1, CSB and CPSF73. WT and *K*_*1268*_*R* CRISPR knock-in (clone E2) cells were either untreated or UV-irradiated with 20 J/m2 and collected 45 min, 24 h and 48 h later. IP was carried out with 4H8 RPB1 antibody.

*K*_*1268*_*R* cells showed little or no growth perturbation under normal conditions, but clonogenic survival assays and growth analysis demonstrated that they are UV-sensitive (Figures 2A–2C and S2A). *K*_*1268*_*R* cells also showed increased sensitivity to treatment with other agents inducing transcription-blocking DNA lesions, such as cisplatin and 4-NQO (Figures S2B and S2C). In yeast, *k1246r* mutation did not give rise to UV-sensitivity (Figure S2D).

When stalled at transcription-blocking DNA lesions, RNAPII acts as a damage sensor, which initiates recruitment of TC-NER factors, most notably the transcription-repair coupling factor CSB, which contains an ubiquitin-binding domain with an important role in TC-NER (Anindya et al., 2010). RNAPII immunoprecipitation from chromatin showed that CSB is efficiently recruited to both WT and K_1268_R RNAPII upon UV irradiation (Figure 2D). In agreement with these data, laser-stripe micro-irradiation revealed that CSB is recruited to sites of DNA damage in both WT and *K*_*1268*_*R* cells (Figures S2E and S2F). These data indicate that the initial step of TC-NER, recruitment of CSB to damage-stalled RNAPII, is largely unaffected by *K*_*1268*_*R* mutation.

While these results show that K_1268_ ubiquitylation is not required for the initial step of TC-NER, it might still affect the overall response to DNA damage, for example through direct or indirect effects on TC-NER rate. To address this possibility, we used a well-established readout of TC-NER efficiency, global recovery of RNA synthesis (RRS) (Mayne and Lehmann, 1982). RRS measurements showed that, after the initial phase of transcription shutdown, both WT and *K*_*1268*_*R* cells generally recovered nascent transcription, in contrast to the CSB-deficient CS1AN cells (Figure 2E). Nevertheless, decreased RRS was observed near the ends of two long genes (*PUM1* and *EXT1*, see Figure S2G) in *K*_*1268*_*R* cells (Figure 2F). Such a reduction was not observed at the medium-length *LMNB1* gene, showing that all lesions in this gene had been repaired at the time of transcription measurement. These data suggest that although TC-NER is not generally defective in *K*_*1268*_*R* cells, they take longer to repair transcription-blocking DNA lesions (Figure 2G).

Rpb1 poly-ubiquitylation/degradation acts as a “last resort” mechanism to remove stalled RNAPII molecules when TC-NER cannot repair the lesion (Wilson et al., 2013a, Woudstra et al., 2002). To test if K_1268_R RNAPII is more persistently stalled at DNA damage, we tracked the kinetics of polymerase removal from chromatin before and after UV irradiation (Figures 2H and S2H). In these experiments, 5,6-dichloro-1-β-D-ribofuranosylbenzimidazole (DRB) was added to cells immediately after UV irradiation, preventing the release of new RNAPIIs into the elongation phase, and allowing us to monitor the fate of RNAPII molecules already engaged in elongation prior to treatment (S_2_-phosphorylated). In the absence of DNA damage, the difference in RNAPII disappearance from chromatin between WT and *K*_*1268*_*R* cells was minimal (Figure 2H, lanes 1–6). In contrast, while WT RNAPII was relatively quickly removed after DNA damage, K_1268_R RNAPII remained chromatin-associated for prolonged periods (Figures 2H, lanes 7–12, and S1E). Interestingly, increased RNAPII retention correlated with markedly increased recruitment and delayed release of CSB, from both chromatin and RNAPII (Figures 2H and S2I). One interpretation of these results is that many damage-stalled RNAPII elongation complexes are normally resolved via degradation (for example so that the lesions in question can be resolved by other means), but that in the absence of RNAPII poly-ubiquitylation and degradation, cells are to a greater extent forced to deal with these complexes through CSB-dependent TC-NER.

### RPB1 Degradation Is a Major Determinant of UV-Induced Shutdown of Transcription

**Figure 3:**
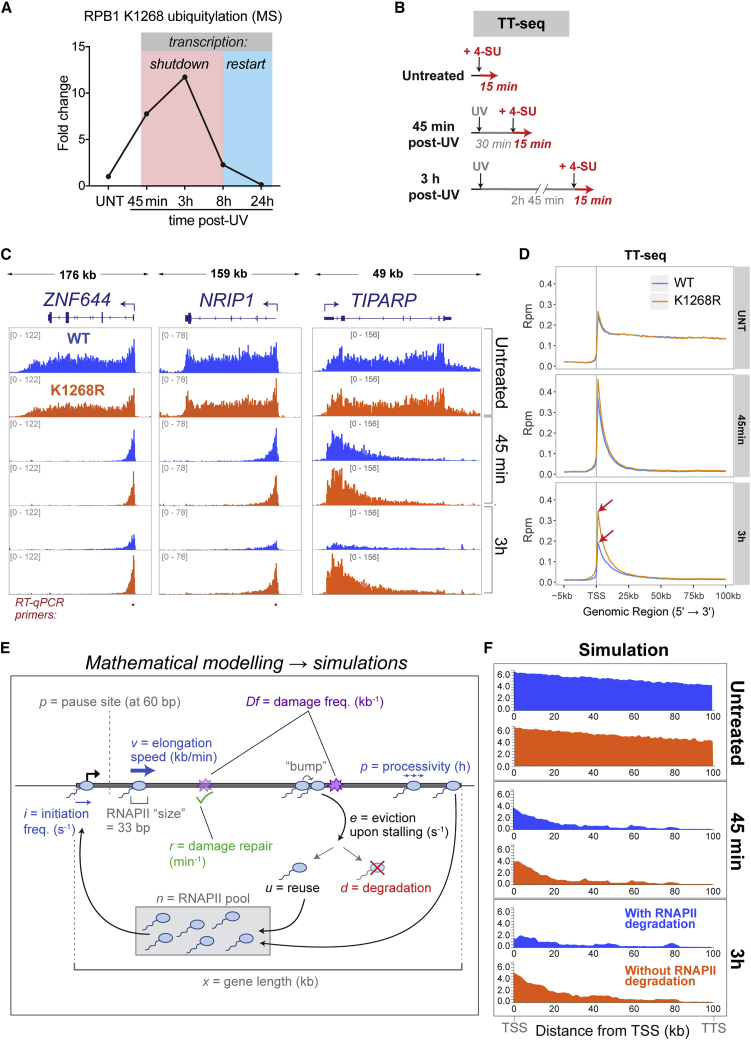
RPB1 Degradation Is a Major Determinant of UV-Induced Shutdown of Transcription Initiation (A) Relative abundance of K_1268_ ubiquitylation, before and at different times after UV irradiation (20 J/m^2^), quantified by TMT Gly-Gly IP mass spectrometry. Data are normalized to untreated controls. Different stages of the transcriptional UV-response are indicated by red and blue boxes. (B) Diagram of experimental design for TT_chem_-seq analysis. (C) Browser tracks from TT_chem_-seq experiment, at *ZNF644*, *NRIP1*, and *TIPARP*. The data are normalized to yeast spike-in. RT-qPCR primers used for validation are indicated below gene panels. (D) Metagene TT_chem_-seq profiles of genes ≧100 kb, in untreated cells and after UV irradiation (20 J/m^2^). Data are normalized to yeast spike-in. TSS, transcription start site. (E) Graphical representation of variables used for *in silico* simulation of RNAPII activity. (F) Simulated RNAPII activity on a 100-kb long gene, before and after DNA damage. RNAPII degradation upon stalling was either allowed (blue panels) or not (orange panels). For all parameter values used, refer to Table S3. See also Figure S3.

**Figure S3:**
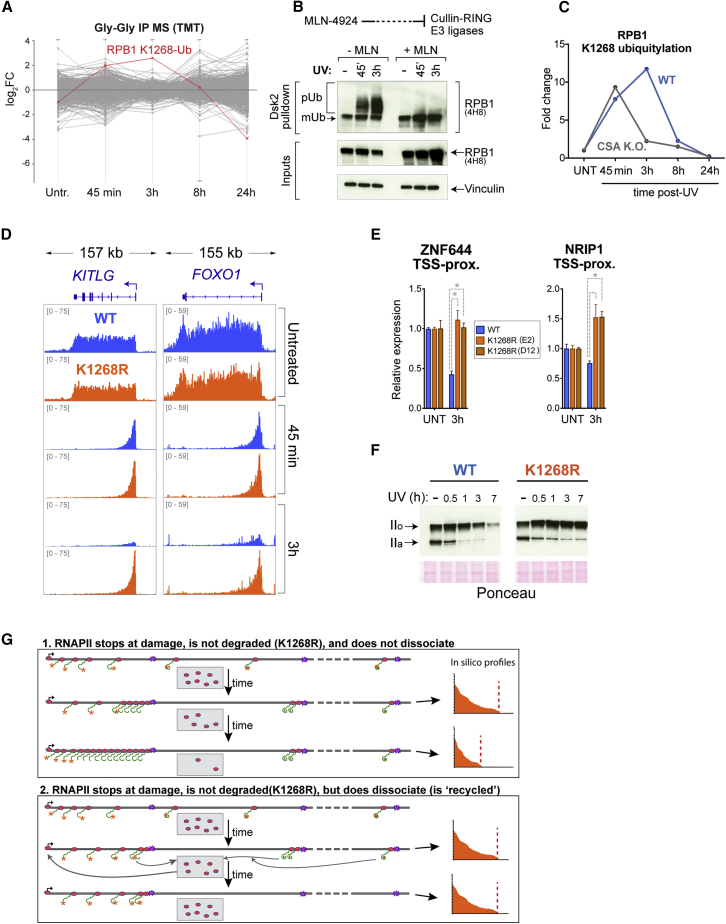
RPB1 Degradation Is a Major Determinant of UV-Induced Shutdown of Transcription Initiation, Related to Figure 3 (A) Abundance of all detected ubiquitylation sites in the proteome of WT cells, at different times after UV irradiation (20 J/m^2^). Each ubiquitylation site is represented as one gray line connecting different time points. K_1268_ ubiquitylation is marked as a red line. Also see Table S2. (B) RPB1 poly-ubiquitylation is inhibited by the NEDDylation inhibitor MLN4924, showing that it requires a cullin E3 ligase (C) K_1268_ ubiquitylation in WT and CSA knock-out cells, before, and at different times after UV irradiation (20 J/m^2^), quantified by TMT Gly-Gly IP mass-spectrometry, normalized to untreated condition. WT only is also shown in Figure 3A. Note that CSA KO cells have normal K_1268_R ubiquitylation at the earliest time-point, strongly indicating that CUL4^CSA^ plays no direct role in it. However, CSA KO cells have defective transcription after UV, potentially explaining why the CSA KO cells have decreased K_1268_ ubiquitylation at this time-point: only transcribing RNAPII is ubiquitylated (Anindya et al., 2007). (D) Browser tracks from the TT_chem_-seq experiment, *KITLG* and *FOXO1* genes. The data are normalized to yeast spike-in. (E) RT-qPCR measuring nascent RNA production at TSS-proximal regions of *ZNF644* and *NRIP1* genes. WT and *K*_*1268*_*R* CRISPR knock-in cells were either untreated, or collected 3 h post UV irradiation (20 J/m^2^). Primer locations are indicated in Figure 3C. Data are normalized to the mature GAPDH expression, and to untreated condition for each cell line. Representative experiments of three biological replicates are shown, with data represented as mean ± SD. Statistically significant differences (p < 0.05, multiple t tests, Holm-Sidak correction) in all three biological replicates are indicated with asterisks. (F) Western blot analysis of chromatin fractions assessing UV-induced RPB1 degradation after 20 J/m^2^ UV irradiation. RNAPII half-life was estimated to be ∼1.5 h in WT cells. (G) Outline of two different scenarios for RNAPII fate at DNA damage when it cannot be degraded (*K*_*1268*_*R* mutant cells), with the predicted transcription activity profiles on the right – in the case where RNAPII dissociation at DNA damage does not take place (upper) or where it does (lower).

Besides DNA repair, the cellular response to transcription-blocking DNA damage involves a dramatic transcriptional response—a rapid and global transcription shutdown, followed by slow recovery (Mayne and Lehmann, 1982, Williamson et al., 2017). Numerous hypotheses have been proposed (e.g., Epanchintsev et al., 2017, Rockx et al., 2000, Vichi et al., 1997), but a definite mechanism underlying this genome-wide phenomenon has remained elusive. Using quantitative ubiquitylation profiling coupled to multiplexing and liquid chromatography-tandem mass spectrometry (LC-MS/MS) after UV irradiation, we realized that K_1268_ ubiquitylation is high in the transcription shutdown phase, and then diminishes to baseline levels during transcription recovery (Figure 3A). RPB1 K_1268_ appears to be the target of the most dramatically UV-induced ubiquitylation event in the whole proteome (Figure S3A; Table S2); it is the target of a Cullin-RING ligase (CRL) (Figure S3B), and its ubiquitylation is affected by, but does not require, CSA (Figure S3C), the targeting subunit of CRL4^CSA^ (Groisman et al., 2003).

To investigate whether K_1268_ ubiquitylation plays a role in transcription, nascent transcription was profiled using a modified transient transcription sequencing (TT_chem_-seq) protocol (Gregersen et al., 2019, Gregersen et al., 2020) (Figure 3B). *K*_*1268*_*R* mutation did not significantly affect nascent transcription in untreated cells, as observed at the level of individual genes (Figure 3C; more gene examples in Figure S3D), and also by metagene-analysis at the genome-wide level (Figure 3D, top panel). The initial response to UV irradiation (at 45 min), when all nascent transcription shifts toward promoter-proximal regions (Williamson et al., 2017), was also highly similar between WT and *K*_*1268*_*R* cells (“elongation shutdown”) (Figures 3C, 3D, and S3D). However, 3 h post-UV, a global decline in nascent RNA levels throughout genes was observed in WT cells (the “initiation shutdown”) (Williamson et al., 2017), while *K*_*1268*_*R* cells retained the same transcription profile as observed 45 min after UV (Figures 3C and 3D, bottom panels, and S3D). The difference between WT and *K*_*1268*_*R* cells was validated by nascent RNA RT-qPCR (Figure S3E). These results further support the idea that UV-induced global transcription shutdown occurs in two stages (Williamson et al., 2017): (1) restriction of transcription elongation to the promoter-proximal 20–30 kb of genes (detected quickly post-UV), and (2) reduction of initiation of new transcription (detected hours post-UV), for which K_1268_ ubiquitylation is important. Without K_1268_ ubiquitylation, transcription initiation thus continues unperturbed.

### Modeling of Transcription after DNA Damage *In Silico*

We next applied a mathematical approach to model RNAPII behavior before and after UV irradiation *in silico*. RNAPII transcription at a model gene was simulated using a mixture of experimentally determined and estimated parameters (Figure 3E; Table S3). We previously showed that such modeling allows the generation of RNAPII “density maps,” which resemble those derived from experiments (Ehrensberger et al., 2013). The model can be tested online at https://github.com/FrancisCrickInstitute/babs_uv_polymerase. In addition to the basic parameters used to model normal steady-state transcription, three parameters were added to simulate transcription after UV irradiation: (1) the DNA lesion frequency after UV irradiation (Mitchell, 1988), (2) the half-life of DNA lesions due to TC-NER (Mellon et al., 1987, Venema et al., 1990), and (3) RNAPII degradation at DNA lesions (Figure S3F). Unexpectedly, the mere inclusion of these three parameters alone was enough to recapitulate, *in silico,* the behavior and kinetics of experimentally derived transcription profiles after UV irradiation, for both WT (i.e., with RNAPII degradation) and *K*_*1268*_*R* cells (without RNAPII degradation) (Figure 3F). Parameters such as general initiation rate, elongation speed, promoter-proximal pausing, etc., had relatively little effect on the overall shape of the *in silico* RNAPII activity profiles, while the introduction of transcription-blocking DNA damage was enough to shift the profiles toward the transcription start site (TSS), suggesting that transcription-blocking DNA damage is the primary constraint that limits RNAPII progression further into genes. This must consequently correspond to the first stage of UV-induced transcription shutdown, observed 45 min after UV (Figure 3F, middle panel). Second, simply allowing the model to integrate degradation of RNAPII when it stalls at a DNA lesion resulted in a decline of simulated transcription 3 h post UV (Figure 3F, bottom panel, blue), therefore faithfully resembling the actual transcription characteristics observed by TT_chem_-seq in WT cells. When RNAPII degradation was inactivated in the computer model, the transcription characteristics of *K*_*1268*_*R* cells were faithfully recapitulated as well (Figure 3F, orange), indicating that the major function of K_1268_ modification is indeed to activate ubiquitin-mediated RPB1 proteolysis. Interestingly, however, to faithfully reproduce *in silico* the unchanging activity profiles observed experimentally between 45 min and 3 h in *K*_*1268*_*R* cells (Figures 3C and S3D), degradation-independent RNAPII dissociation from sites of DNA damage had to be introduced. Indeed, without such dissociation and recycling, RNAPII molecules would quickly pile up head-to-tail at DNA damage and shift the activity profiles of *K*_*1268*_*R* cells closer and closer to the promoter over time (Figure S3G). It would also effectively deplete RNAPII for use in initiation at other genes so that transcription would shut down in these cells as well, which was not observed.

We were surprised by these findings, given the multiple models proposed to explain transcription shutdown after UV irradiation (Adam et al., 2013, Dinant et al., 2013, Epanchintsev et al., 2017, Mourgues et al., 2013, Oksenych et al., 2013, Proietti-De-Santis et al., 2006, Williamson et al., 2017). Our measurements of nascent transcription (Figures 3C, 3D, and S3D) alongside the computer modeling above indicate that initiation of transcription becoming inhibited correlates with degradation of RPB1 (Figures 1F and S3F), and *K*_*1268*_*R* cells with non-degradable RNAPII actually continue to initiate new transcription after UV. Altogether, the results with *K*_*1268*_*R* mutant cells thus provide a simple explanation for the shutdown of transcription after DNA damage: (1) DNA lesions impose a physical constraint for transcript elongation to proceed much beyond the TSS-proximal 20–30 kb region, and (2) cells deplete RNAPII levels in accordance with the level of DNA damage, so that when RNAPII levels are sufficiently low, it becomes limiting and transcriptional initiation ceases.

### K_1268_ Ubiquitylation Prevents Short Genes from Escaping Transcription Shutdown

**Figure 4:**
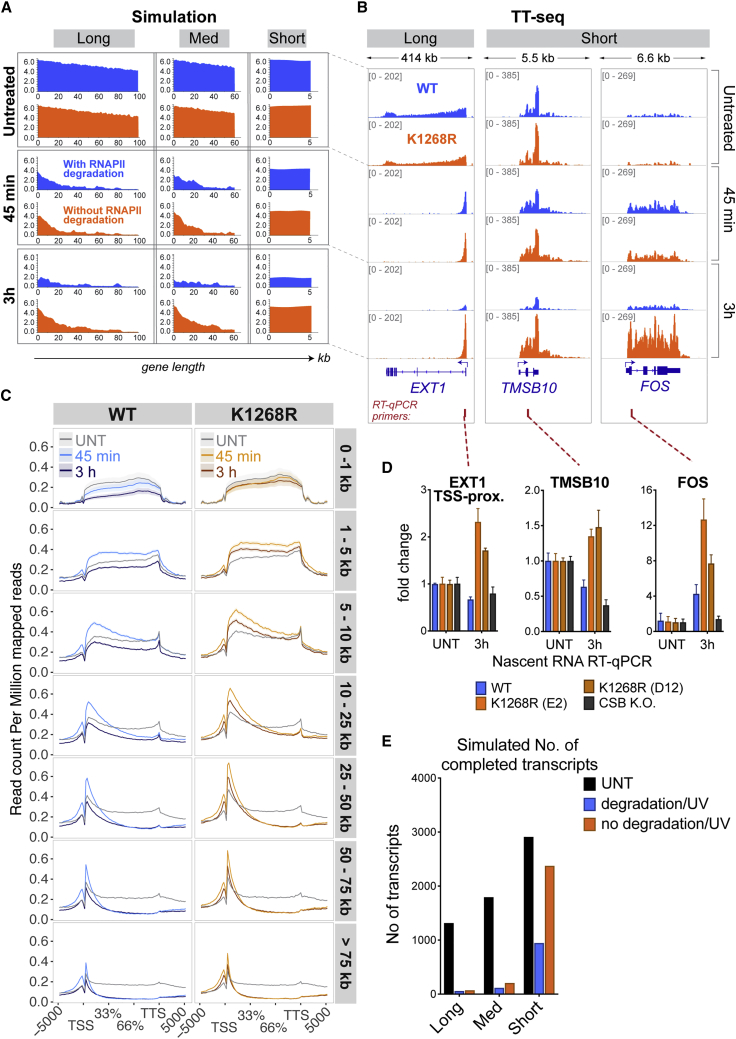
K_1268_ Ubiquitylation Ensures that Short Genes Also Cease Expression upon UV Irradiation (A) Simulated RNAPII activity on a long, medium, and short gene, with or without DNA damage. Three genes are competing for the same pool of RNAPII molecules. The initiation probability was weighted by the relative representation of long (>100 kb), medium (30–100 kb), and short (<30 kb) genes in the genome (0.1: 0.2: 0.7, respectively). RNAPII degradation upon stalling was either allowed (blue) or not (orange). (B) Browser tracks of TT_chem_-seq data, from a long (*EXT1*) and two short genes (*TMSB10* and *FOS*). The data are normalized to yeast spike-in. RT-qPCR primers used for validation are indicated below gene panels. (C) Metagene TT_chem_-seq profiles of all genes in the genome, stratified by gene length (indicated in bold on the right). x axis: relative scale (TSS and TTS are indicated); y axis: reads per million mapped reads (rpm). Transcription levels in untreated cells (gray lines), and 45 min (light-colored lines) and 3 h (dark-colored lines) after UV irradiation (20 J/m^2^) are shown. The data are normalized to yeast spike-in. (D) Nascent RNA production after UV irradiation (20 J/m^2^) at TSS-proximal regions of *EXT1*, *TMSB10*, and *FOS* genes. RT-qPCR primer positions are indicated in (B). Data are represented as mean ± SD and normalized to the mature GAPDH transcript and to untreated conditions. (E) Simulation-predicted number of mRNA transcripts in a long, medium, and short gene, in untreated cells and 4 h post-damage, in scenarios where RNAPII degradation is allowed (WT equivalent) or not (K_1268_R equivalent). Parameter values as in (A). See also Figure S4 and Table S3.

**Figure S4:**
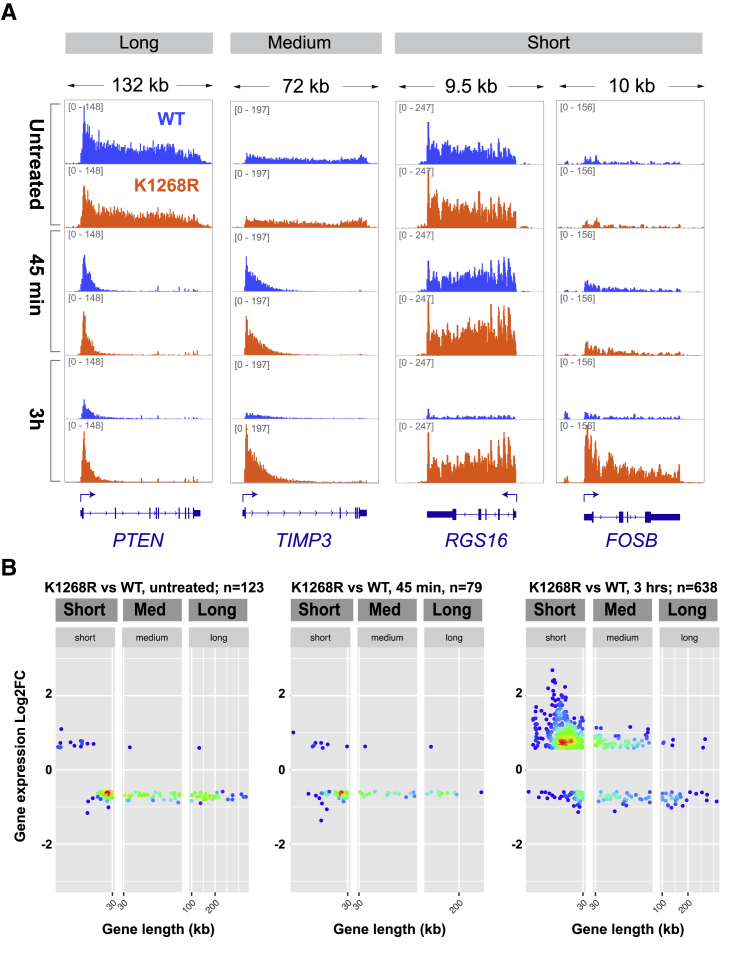
K1268 Ubiquitylation Prevents Short Genes from Escaping the UV-Induced Transcription Shutdown, Related to Figure 4 (A) Browser tracks of the TT_chem_-seq experiment, showing a long (*PTEN)*, a medium (*TIMP3*) and two short genes (*RGS16 and FOSB*), before and 45 min or 3 h after UV irradiation. The data are normalized to yeast spike-in. (B) Scatter-density plots showing the genes that are differentially expressed between *K*_*1268*_*R* and WT cells (TT_chem_-seq data) stratified by gene length, at different times after UV irradiation (20 J/m^2^). Each gene is represented by one dot. Plots are colored by binned spot density from low (blue) to high (red).

Considering that UV-induced DNA damage restricts nascent transcription to an area 20–30 kb downstream of the transcription start site, we hypothesized that short genes might represent a distinct group of genes, as also suggested by previous work (McKay et al., 2004, Williamson et al., 2017). We therefore computer-simulated the competition for the same limited pool of RNAPII by 3 different gene types: short (arbitrarily chosen as = 5 kb; the precise length of these model genes is not important), medium (63 kb), and long (100 kb) (Figure 4A; Table S3). Simulation *in silico* predicted that in WT cells, all gene types will suffer transcription shutdown, while short genes escape such shutdown in *K*_*1268*_*R* cells (i.e., in the absence of RNAPII degradation) (Figure 4A, bottom right panel). To experimentally test these predictions, we investigated individual genes, as well as global TT_chem_-seq profiles by stratifying by gene length, and observed that the predictions made *in silico* were correct (Figures 4B–4D, S4A, and S4B). Similarly, we counted the number of mRNA transcripts that were predicted to be produced during the simulation. With RNAPII degradation disabled, the abundance of mRNA transcripts arising from short genes was predicted to be relatively unchanged upon DNA damage within the simulated time frame (4 h post-UV), while long and medium transcripts were downregulated (Figure 4E). Interestingly, our experiments showed that some genes, including the proto-oncogene *FOS*, showed marked upregulation upon UV irradiation (transient increase in WT, persistent increase in *K*_*1268*_*R* mutant), which was detected both by TT_chem_-seq and nascent RNA RT-qPCR (Figures 4B and 4D). This indicates that, not surprisingly, a subset of genes is DNA damage-induced, and that RNAPII degradation prevents sustained expression of such short genes upon UV irradiation in WT cells.

**Figure 5:**
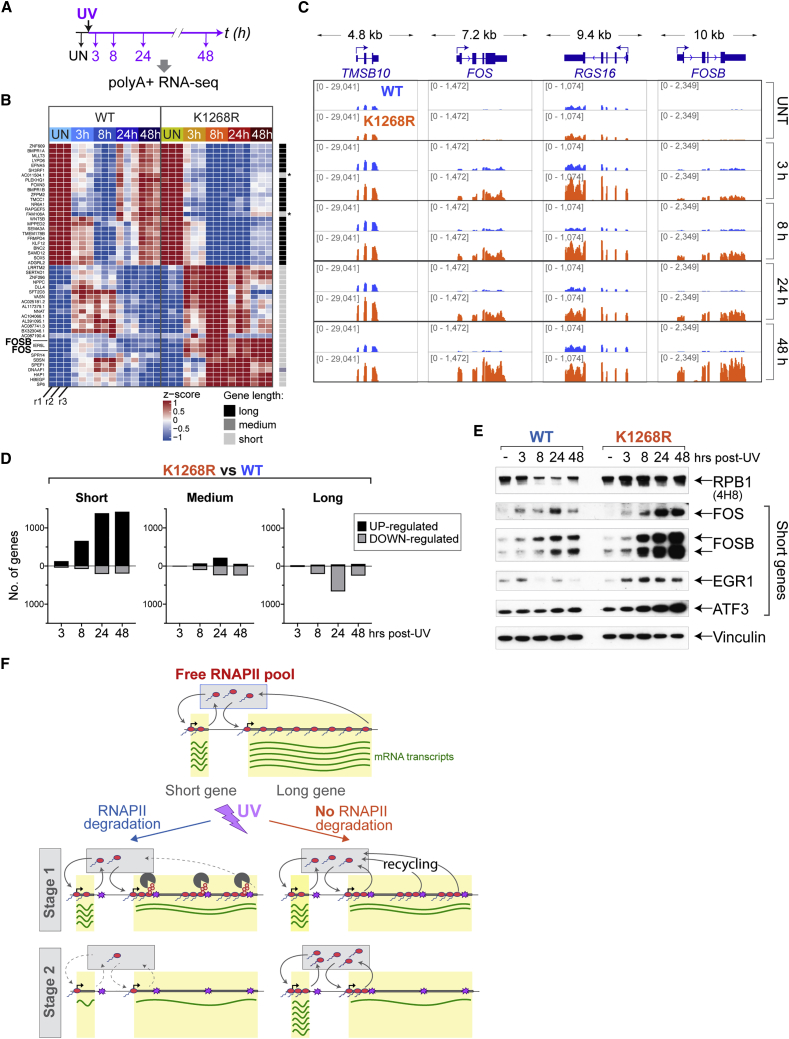
K_1268_ Ubiquitylation Is Required to Prevent Long-Term Transcriptional Defects upon Acute Exposure to UV (A) Experimental design. (B) Heatmap, showing expression over time, of the top 50 down- and upregulated genes in *K*_*1268*_*R* cells 8 h after UV irradiation (20 J/m^2^). Each column within a treatment group represents a biological replicate (r1, r2, r3), and each row represents one gene. Gene lengths are shown on the right, in shades of gray. Asterisks indicate genes mis-annotated as short, but confirmed by manual inspection to be long. (C) Browser tracks from mRNA-seq experiment at four short genes. (D) Bar plots of genes differentially expressed between *K*_*1268*_*R* and WT cells (logFC >1, false discovery rate [FDR] <0.01), for short, medium, and long genes, at different times after UV irradiation (20 J/m^2^). Positive side of y axis: upregulated genes; negative side: downregulated genes. (E) Western blot showing proteins encoded by short, immediate-early genes, at different time points after UV irradiation (20 J/m^2^). (F) Model depicting how DNA damage and RNAPII (red sphere) levels influence transcription and mRNA level (green lines) after UV irradiation, in the first stage (45 min equivalent, middle panel) and second stage (3 h + equivalent, bottom panel) of the transcription shutdown. See also Figure S5.

**Figure S5:**
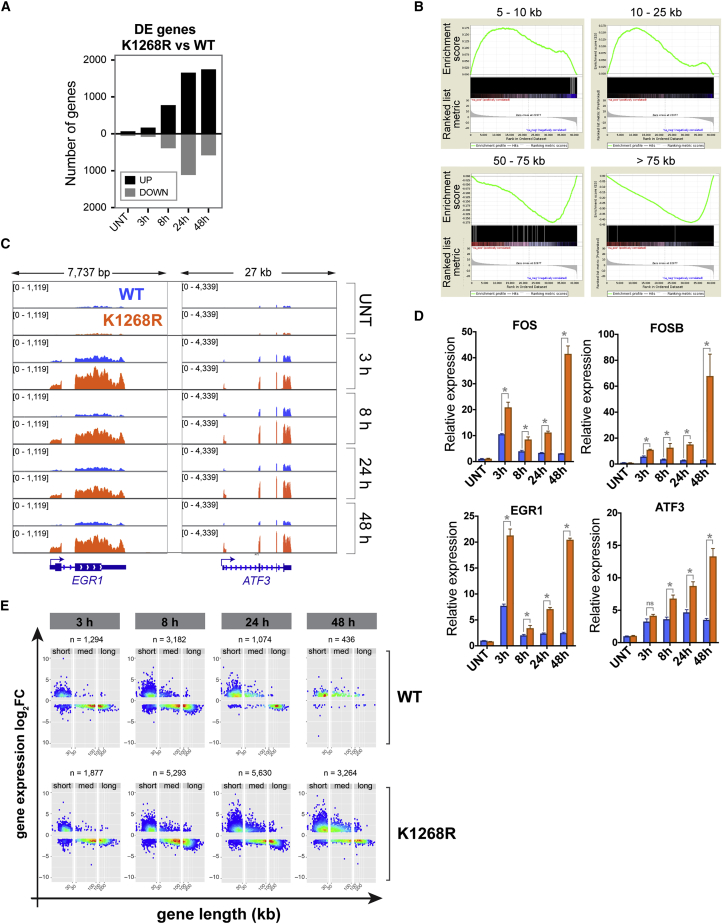
K1268 Ubiquitylation Is Required to Prevent Long-Term Transcriptional Defects upon Acute Exposure to UV, Related to Figure 5 (A) Number of differentially expressed genes (mRNA-seq) between *K*_*1268*_*R* and WT cells, at different time points after UV irradiation (20 J/m^2^). Black bars: upregulated genes; gray bars: downregulated genes. (B) Gene set enrichment analysis showing the enrichment of short (top two panels) and medium to long genes (bottom panels) in the differentially expressed gene datasets (24 h) between K_1268_R versus WT. (C) Browser tracks of the RNA-seq experiment, showing the expression of two short genes (*EGR1* and *ATF3*). (D) RT-qPCR, measuring the abundance of mature, poly-adenylated transcripts of four short genes (FOS, FOSB, EGR1 and ATF3), in WT and *K*_*1268*_*R* cells, at different times after UV irradiation (20 J/m^2^). Data are normalized to GAPDH and untreated condition. A representative experiment of three biological replicates is shown; data are represented as mean ± SD. Asterisks indicate statistically significant differences in all three biological replicates (p < 0.01, multiple t tests, Holm-Sidak correction). (E) Scatter-density plots showing the UV-regulated genes in the mRNA-seq data (differentially expressed genes between each UV-treated condition and untreated condition, logFC > 1, FDR < 0.01), for K_1268_R and WT cells separately. The total number of differentially expressed genes (n) in each condition is indicated on top of the plots. Genes were stratified by gene length (short: < 30 kb; medium: 30-100 kb; long: > 100 kb), and each gene is represented by one dot. Plots are colored by binned spot density from low (blue) to high (red).

Profiling of stable, poly-adenylated mRNA transcripts in WT and *K*_*1268*_*R* cells by mRNA sequencing (mRNA-seq) at different time points after UV irradiation revealed that the number of differentially expressed genes (DEGs) between *K*_*1268*_*R* mutant and WT increases over time, reaching a peak 24 h post-UV (Figure 5A); this was particularly pronounced for DEGs upregulated in the *K*_*1268*_*R* mutant (>1,600 genes), which continued to accumulate even 48 h post-UV (Figure S5A). An unsupervised analysis of the top 50 DEGs revealed that virtually all upregulated genes (in *K*_*1268*_*R* mutant compared to WT) are short, while downregulated genes are invariably long (Figure 5B). This strong tendency was confirmed by gene set enrichment analysis of the entire DEG dataset (Figure S5B).

The extensive and sustained upregulation of short genes in the *K*_*1268*_*R* mutant was unmistakable also when individual gene examples were examined (Figures 5C, S5C, and S5D) or when UV-regulated genes were plotted separately in WT and *K*_*1268*_*R* mutant cells (Figure S5E), revealing that more than 1,000 short genes were affected at the mRNA level. Direct comparison of UV-regulated genes confirmed the strong upregulation of short genes in the *K*_*1268*_*R* mutant (Figure 5D). We conclude that *K*_*1268*_*R* cells accumulate mRNAs of thousands of short genes upon UV exposure due to their inability to degrade RNAPII. For all the examples we investigated, the increase in short-gene mRNAs was translated into more protein upon UV irradiation (Figure 5E). Importantly, many of the most highly expressed short genes are immediate-early genes (IEGs), which often encode transcription factors and oncoproteins (Tullai et al., 2007), and may thus reinforce the aberrant gene expression program in *K*_*1268*_*R* cells, even at late stages of the UV response where their levels remained markedly higher than even the UV-induced level in WT cells (Figure 5E, compare the 8, 24, and 48 h time points). Overall, these results demonstrate that an inability to degrade RNAPII upon UV irradiation results in extreme dysregulation of transcription, including erratic induction of a large number of predominantly short gene transcripts that are translated into proteins, many of which are oncoproteins. These results emphasize the crucial role played by the overall cellular RNAPII pool, a decrease of which allows cells to avoid profound and permanent dysregulation of transcription upon exposure to transcription-blocking DNA damage (Figure 5F).

### Regulation of Global RNAPII Levels Underlies Transcription Recovery upon DNA Damage

**Figure 6:**
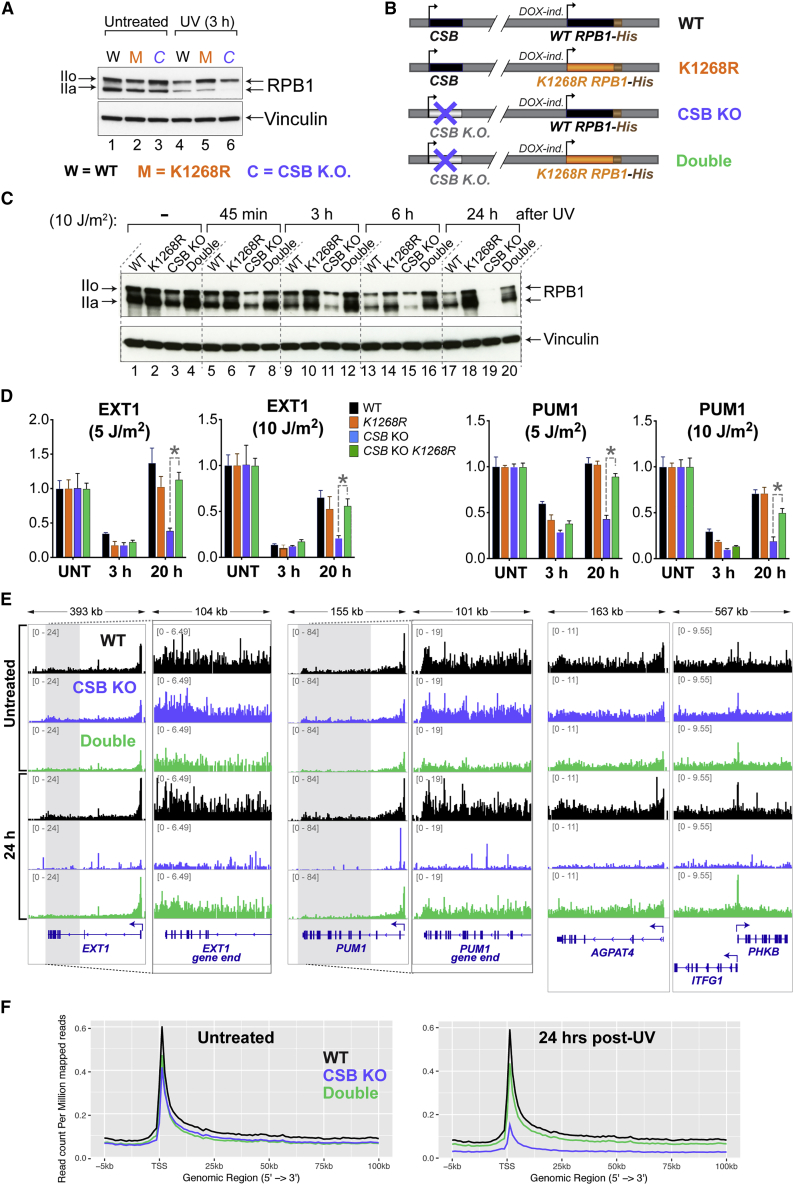
RPB1 Stability Determines Transcription Recovery upon UV Irradiation (A) Western blot showing the total levels of RPB1 before and after UV irradiation (10 J/m^2^). (B) Diagram of cell lines. (C) As in (A), but using the cell lines from (B) and testing later time points. (D) RT-qPCR measuring nascent RNA production at the end of the long *EXT1* and *PUM1* genes at different times after different UV doses. Data are normalized to the expression of mature GAPDH transcript, and to untreated conditions, and represented as mean ± SD. Statistically significant differences (p < 0.05, multiple t tests, Holm-Sidak correction) in all three biological replicates are indicated with asterisks. (E) Browser tracks from TT_chem_-seq experiments. The data are normalized to yeast spike-in. (F) Metagene TT_chem_-seq profiles. Data are normalized to yeast spike-in. See also Figure S6.

Defective TC-NER causes an increase in RNAPII stalling at DNA lesions, and persistent RNAPII stalling triggers RNAPII poly-ubiquitylation and degradation (Somesh et al., 2005, Wilson et al., 2013a, Woudstra et al., 2002). Therefore, it might be expected that RNAPII poly-ubiquitylation/degradation would increase in the absence of TC-NER, thus affecting overall RNAPII levels. In agreement with this idea, we observed somewhat lower RPB1 levels 3 h after UV irradiation in *CSB* knockout (KO) cells compared to isogenic WT control cells (Figure 6A, compare lanes 4 and 6). Others have observed such a decrease as well (He et al., 2017, Proietti-De-Santis et al., 2006).

**Figure S6:**
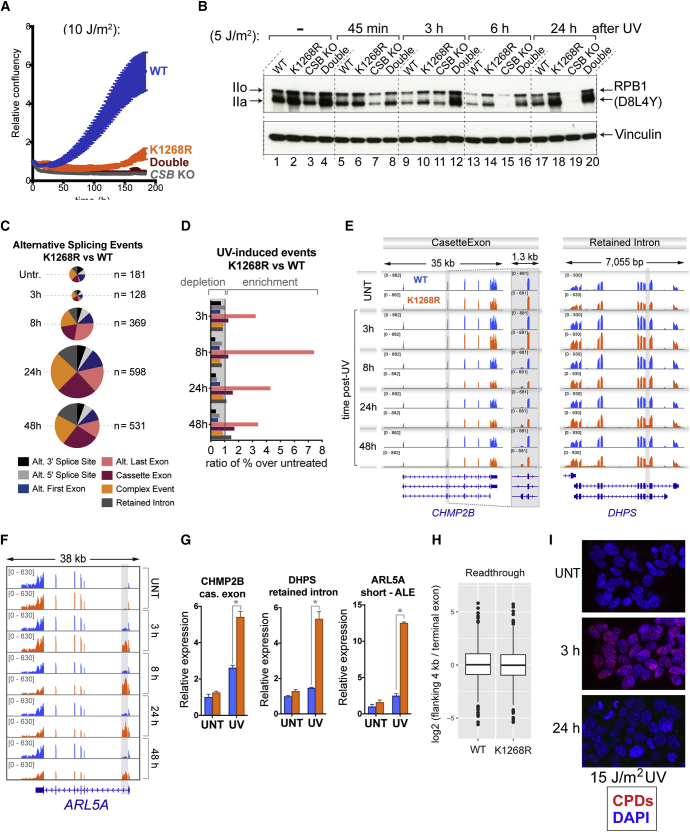
Other Effects of K_1268_R Mutation, Related to Figure 6 and Discussion (A) Growth assays before and after UV irradiation (10 J/m^2^), in switchover model cell lines represented in Figure 6B. Cell growth (confluency) was monitored every 3 h after UV irradiation using Incucyte and the data were normalized to t = 0 for each well. Data are represented at each 3h time point as average relative confluency of 3 biological replicates ± SD (B) As in Figure 6C, but with 5 J/m^2^ UV irradiation. (C) Alternative splicing differences between *K*_*1268*_*R* and WT cells, at different time points after UV irradiation (20 J/m^2^), detected in the mRNA-seq data. Pie-chart categories show the proportions of different classes of alternative splicing events. The size of the pie-charts is proportional to the total number of differences (n, indicated on the right). (D) Enrichment of differential splicing events (*K*_*1268*_*R* versus WT) at different time points after UV irradiation. Enrichment was calculated by comparing the proportion of each class of events in the given UV-treated condition, to the proportion of the same class in untreated condition. (E and F) Browser tracks of the RNA-seq experiment, showing the examples of three genes (*ARL5A, CHMP2B* and *DHPS)* with alternative splicing events induced by UV irradiation preferentially in *K*_*1268*_*R* cells. (G) RT-qPCR measuring the abundance of alternatively spliced poly-adenylated transcripts in WT and K_1268_R cells, in untreated condition and 24 h after UV irradiation (20 J/m^2^). The data were normalized to the expression of the mature GAPDH transcript and untreated condition. A representative experiment of three biological replicates is shown, data are represented as mean ± SD. Asterisks indicate statistically significant differences in all three biological replicates (p < 0.01, multiple t tests, Holm-Sidak correction). (H) Analysis of transcription readthrough beyond the TTSs. Ratios of read-counts of the 4kb region downstream of the TTS and the terminal exon of all protein coding and RNA genes, derived from TT_chem_-seq experiment, are plotted for WT and *K*_*1268*_*R* cells, in untreated conditions. (I) Immunofluorescence detection of CPDs in WT HEK293 cells, 3 h and 24 h after exposure to 15 J/m^2^ of UV irradiation.

To test if the lack of transcription recovery in TC-NER-deficient CSB KO cells might be at least partly attributed to the effect on RNAPII stability, we generated a double *K*_*1268*_*R CSB* KO cell line, with appropriate control cells (Figure 6B). Not surprisingly, given that the *K*_*1268*_*R* and *CSB* KO cells are individually UV-sensitive, the double-mutated cells were UV-sensitive as well (Figure S6A). RPB1 stability was assessed in these cells at different times following irradiation with different UV doses (5 J/m^2^ and 10 J/m^2^; markedly lower than used in the experiments of Figures 1, 2, 3, and 4). This first revealed that, in the *CSB* KO, not only is RPB1 degraded faster during transcription shutdown (45 min, 3 h, 6 h), but—more importantly—RPB1 levels failed to recover in the transcription restart phase (24 h) (Figure 6C, compare lanes 17 and 19; see also Figure S6B for 5 J/m^2^). UV-induced RPB1 degradation was largely prevented in *CSB* KO cells carrying the *K*_*1268*_*R* mutation, with RPB1 levels remaining relatively stable over time (Figure 6C, compare lanes 17 to 20; see also Figure S6B). These results suggest that the absence of CSB causes markedly reduced RPB1 stability due to increased K_1268_ ubiquitylation and proteasomal degradation.

We now investigated the consequences for transcription recovery of stabilizing RNAPII in *CSB* KO cells. Strikingly, nascent RNA RT-qPCR measurements of two individual long genes (*EXT1* and *PUM1*) demonstrated that, in sharp contrast to *CSB* KO cells, *CSB* KO cells carrying *K*_*1268*_*R* mutation are able to recover transcription after UV irradiation (Figures 6D). *K*_*1268*_*R* mutation appears to cause a delay in DNA damage repair (Figure 2F), which may explain the difference in transcription recovery between WT and double mutant cells.

To further expand on the surprising difference between *CSB* KO cells and the double-mutated cells, we compared nascent transcription by TT_chem_-seq in these cells 24 h after 5 J/m^2^ UV irradiation. This analysis showed that *K*_*1268*_*R* mutation generally salvaged nascent transcription in *CSB* KO cells, as observed both at the level of individual genes (Figure 6E) where it was most clearly observed near gene-ends (Figure 6E, panels 2 and 4 from the left), but also by metagene-analysis (Figure 6F). Together, these results indicate that the lack of transcription recovery upon DNA damage in cells lacking Cockayne syndrome B is primarily due to decreased RNAPII stability in these cells.

## Discussion

**Figure 7:**
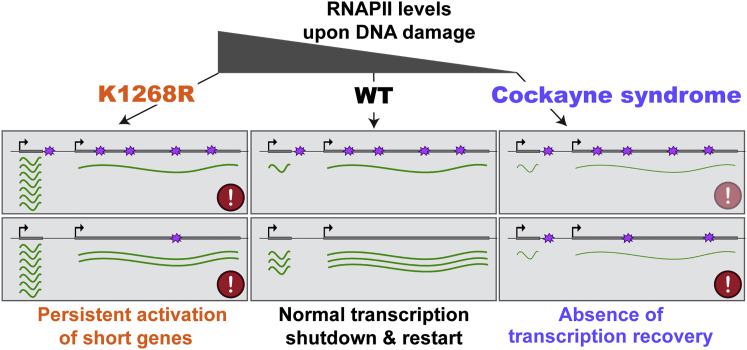
A Model for Transcription and Its Global Control by the Free RNAPII Pool Simplified model, illustrating how RNAPII levels in WT, *K*_*1268*_*R*, and Cockayne syndrome cells regulate the global transcriptional response to UV irradiation. mRNA, green lines; DNA lesions, purple stars. Top panels denote transcriptional shutdown, bottom panels restart. Repair of transcription-blocking DNA lesions is slower in *K*_*1268*_*R* cells, and little TC-NER occurs in *CSB* cells, partly because CSB protein is required for it, but also because TC-NER requires RNAPII, which is depleted in these cells. Combined with other mechanisms (e.g., Epanchintsev et al., 2017), such depletion means that transcription shutdown is more rapid in CSB cells (shown as thin green lines).

In this report, we show that a single ubiquitylation site in RPB1 (K_1268_) regulates DNA damage-induced degradation of RNAPII in human cells, and this process is essential for cell survival after genotoxic stress. K_1268_ ubiquitylation affects the global transcriptional response to UV irradiation. Indeed, RPB1 K_1268_ ubiquitylation is required to regulate the size of the overall RPB1 pool, which in turn determines the capacity of cells to initiate new transcription. Suppression of transcriptional initiation is required to avoid aberrant expression of thousands of short genes, which are otherwise relatively permissive for continued, high-level transcription upon DNA damage. We speculate that these genes, many of which are damage-induced immediate-early genes and oncogenes (Tullai et al., 2007), need to be turned off again for cells to survive acute genotoxic stress. At the other end of the spectrum, abnormal depletion of the RNAPII pool upon DNA damage is observed in cells lacking CSB. Unexpectedly, the depletion of RNAPII long after UV irradiation is actually almost solely responsible for the lack of transcription recovery in *CSB* KO cells. Together, these results indicate that tight regulation of overall RNAPII levels is critical for correct transcriptional regulation upon DNA damage (Figure 7).

### A Single Poly-ubiquitylation Site as a Signal for RPB1 Degradation

Poly-ubiquitin chains that target proteins for proteasomal degradation are most often conjugated to the substrate without absolute site specificity (i.e., if the “normal” site of ubiquitylation is mutated, lysines in close proximity can typically act as alternative acceptor sites) (Mattiroli and Sixma, 2014). In this regard, RPB1 K_1268_ presents an unusual case, being the dominant or only site for RPB1 poly-ubiquitylation. Ubiquitylation at this site, and its singular effect on ubiquitylation/degradation, is conserved from yeast to humans. Notably, mono-ubiquitylated RPB1 is still detectable in *K*_*1268*_*R* mutant cells, and might represent modification at one or more other residues, each detected as ubiquitylated at markedly lower levels than K_1268_ (Table S1). Previous experiments on the mechanism of RNAPII ubiquitylation and degradation in yeast and human cells indicate a complex sequence of events, where NEDD4 (Rsp5 in yeast) first mono-ubiquitylates, and Elongin_ABC_-CUL5 (Ela1-Elc1-Cul3 in yeast) then poly-ubiquitylates RPB1 (Harreman et al., 2009, Wilson et al., 2013a, Wilson et al., 2013b). CUL2^VHL^ may contribute to this process as well (Kuznetsova et al., 2003). While preventing poly-ubiquitylation by mutation of other RPB1 sites has not proven possible (A.T.V. and M.N., unpublished data), possibly because alternative, nearby sites are being modified in their absence, knowledge of these RPB1 ubiquitylation sites and the dramatic effect of *K*_*1268*_*R* mutation will hopefully now allow a more detailed mechanistic investigation of RPB1 ubiquitylation.

It is worth emphasizing that the complex transcriptional consequences of *K*_*1268*_*R* mutation after DNA damage can be faithfully recapitulated *in silico* merely by applying the assumption that such mutation prohibits RNAPII degradation, strongly indicating that this is its major role. Indeed, although we obviously cannot completely rule out that K_1268_ ubiquitylation might affect transcription in other ways as well, these would likely be secondary/minor compared to the effect of *K*_*1268*_*R* mutation on RNAPII degradation.

The data presented here arguably also allow a better interpretation of the changing RPB1 phosphorylation states observed after UV irradiation. The (unphosphorylated) II_A_ form represents the free/initiating RNAPII form, while the (hyper-phosphorylated) II_0_ form represents elongating polymerases, unavailable for new transcription initiation as they are already engaged in transcription. Upon UV irradiation, polymerases start transcription (and become hyper-phosphorylated), reach a DNA lesion, and are degraded. This II_A_ → II_0_ → degradation cycle is repetitive unless DNA lesions are removed, and eventually leads to cells starting to run out of free (II_A_) polymerase, as observed in WT cells 3 h post-UV. Without free polymerase, no new transcription can initiate. In the *K*_*1268*_*R* mutant, RNAPII degradation does not take place and damage-stalled polymerases are recycled and returned to the free/initiating pool, so the II_A_ form is never completely depleted, allowing these cells to continue transcription initiation. Elongating (II_0_) polymerases in *K*_*1268*_*R* cells stay on chromatin longer (and thus accumulate) as they are delayed/stopped by DNA damage, but they are not degraded.

### RPB1 K_1268_ Ubiquitylation Affects DNA Repair

For TC-NER to take place, stalled RNAPII has to somehow be removed from the DNA lesion to allow access for the DNA repair machinery. This might be achieved by RNAPII backtracking or displacement (Gregersen and Svejstrup, 2018). It has also been suggested that removal of damage-stalled RNAPII by ubiquitylation and proteasomal degradation might enable TC-NER (Bregman et al., 1996). However, studies in yeast show that Rpb1 ubiquitylation/degradation is *not* required for TC-NER (Lommel et al., 2000, Woudstra et al., 2002). Here, it is relevant to note that RNAPII is still efficiently displaced from DNA lesions in human *K*_*1268*_*R* cells. Indeed, we found that RNAPII activity profiles remain constant between 45 min and 3 h after inducing DNA damage in these cells, meaning that RNAPII is constantly recycled from damage sites even in the absence of ubiquitylation/degradation. This finding is important. Indeed, the extraordinary stability of RNAPII elongation complexes (Gnatt et al., 1997, Gnatt et al., 2001), combined with the inability of purified Rad26/CSB to displace RNAPII from a DNA lesion *in vitro* (Selby and Sancar, 1997, Xu et al., 2017) has led to the generally accepted view that RNAPII encountering a DNA lesion remains on DNA for extended periods of time (Gregersen and Svejstrup, 2018, Hanawalt and Spivak, 2008, Vermeulen and Fousteri, 2013). Our data suggest that this is incorrect; at the very least, RNA polymerases do not pile up behind each other at DNA lesions to an extent that leads to a measurable depletion of the free RNAPII pool, which might otherwise be expected in the absence of RPB1 degradation. Moreover, even though stalled RNAPII can be detected at DNA damage for at least 48 h in TC-NER defective cell lines (Garinis et al., 2009), we posit that it cannot be the same polymerase molecules that are detected, but that there is instead constant RNAPII turnover at unrepaired DNA lesions. The mechanism of RNAPII dissociation remains unclear, and whether displacement is focused on the lesion-stalled polymerase itself or the polymerases piling up behind it also remains to be discovered. The *K*_*1268*_*R* cell line should make it possible to uncover the factors required for RNAPII dissociation and recycling during DNA damage.

### RPB1 K_1268_ Ubiquitylation Is Required for the Global Transcriptional Response to UV Irradiation

It has been known for decades that UV irradiation elicits a complex transcriptional response, involving rapid and global transcription shutdown, followed by subsequent transcription recovery 10–24 h after UV exposure (Mayne and Lehmann, 1982). Transcription shutdown occurs in two stages: first, an almost instantaneous effect on transcript elongation (Lavigne et al., 2017, Williamson et al., 2017), and second, global inhibition of transcriptional initiation (Gyenis et al., 2014, Proietti-De-Santis et al., 2006, Rockx et al., 2000, Williamson et al., 2017). Several mechanisms have been proposed to explain UV-induced transcription shutdown and restart, most of which involve factors that regulate RNAPII activity in *trans* (Epanchintsev et al., 2017, Kristensen et al., 2013, Rockx et al., 2000, Vichi et al., 1997). For example, it has been suggested that UV-induced stabilization of the general transcription repressor ATF3 is responsible for global transcription shutdown and for the lack of transcription recovery after UV exposure (Epanchintsev et al., 2017, Kristensen et al., 2013). The results presented here effectively rule out the possibility that this mechanism underlies the shutdown. First, if ATF3 were indeed responsible for transcription shutdown, *K*_*1268*_*R* cells (that lack transcription initiation shutdown) would be expected to lack ATF3. Instead, ATF3 is markedly up-regulated upon UV irradiation in these cells (see Figure 5E). Second, the introduction of *K*_*1268*_*R* mutation alone is sufficient to rescue the transcription recovery defects in *CSB*-deficient cells, again strongly arguing that RPB1 stability, and not ATF3-mediated transcriptional repression, is the primary determinant of the transcriptional response to DNA damage in these cells as well.

Modern genomics techniques typically provide only single time point snapshots of RNAPII activity. Our findings highlight the potential of mathematical modeling and *in silico* simulation to explain the transcription dynamics that underlie such data. In the case presented here, simulation helped simplify what appeared to be a highly complex process. Actually, UV-induced transcription profiles can be explained solely by the frequency of DNA damage in the genome, and by RPB1 degradation. When there is frequent DNA damage, the RNAPII pool becomes depleted, so that new initiation is prohibited: transcription shuts down. When the RNAPII pool recovers to normal levels, transcription resumes.

Modeling *in silico* also predicted that a lack of RPB1 degradation will cause an uneven DNA damage-induced transcription shutdown across gene classes, depending on length. Indeed, thousands of short genes escape the UV-induced transcription shutdown if damage-induced RNAPII degradation does not take place. Dominant among these are the so-called immediate-early genes (IEGs), which are rapidly but transiently upregulated following a variety of stimuli, such as growth factors, hormones or cellular stress (Bahrami and Drabløs, 2016). Interestingly, almost all stress-responsive genes, including the IEGs, are short, allowing them to be highly expressed in response to UV irradiation. We speculate that these genes evolved to be short so they can be upregulated upon UV irradiation, one of the most ancient cellular stresses. Many IEGs are proto-oncogenes, whose sustained expression drives cellular growth and transformation (Fowler et al., 2011, Healy et al., 2013). The IEGs thus need to be turned off after the initial need for their expression has subsided. Such shutdown does not occur in *K*_*1268*_*R* mutant cells, and we speculate that these cells are UV-sensitive at least partly because they dysregulate a wide array of growth-regulating genes at a time when they should stop the cell cycle and focus on repairing DNA damage.

It was previously observed that *K*_*1246*_*R* mutation affects mRNA splicing in yeast (Milligan et al., 2017). Possible consequences for splicing kinetics remain to be investigated in detail, but our analysis of RNA sequencing (RNA-seq) data indicated little or no effect of *K*_*1268*_*R* mutation on intron retention, and only very limited effects on the final splicing outcomes were observed in the absence of UV irradiation in human cells (Figures S6C–S6G, and data not shown). Similarly, based on experiments in yeast, it was proposed that ubiquitylation and proteasomal degradation of RNAPII might play a role in transcriptional termination (Gillette et al., 2004). No noteworthy effect of *K*_*1268*_*R* mutation on transcriptional termination was observed in human cells (Figure S6H, and data not shown). Conversely, while human *K*_*1268*_*R* cells are UV-sensitive, yeast *RPB1 K*_*1246*_*R* cells are not. We note that, similarly, human *CSB*-deficient cells are UV-sensitive, but yeast cells lacking *RAD26* (encoding the CSB homolog) are not. Together, these data indicate some divergence between yeast and humans in the physiological consequence of lacking RPB1 ubiquitylation or TC-NER. The reasons for these differences remain a matter of speculation, but we note that yeast genes are invariably very short, which might affect the need for regulating gene expression through RPB1 stability.

### RPB1 Stability Determines Transcription Recovery upon UV Irradiation

An almost complete lack of transcription recovery after UV irradiation is the hallmark of Cockayne syndrome cells (Mayne and Lehmann, 1982). It has been assumed that the failure to re-start transcription is due to defective TC-NER, i.e., that DNA lesions in the transcribed strand of genes cannot be repaired without the activity of transcription-repair coupling factor CSB. However, the results presented here establish that, actually, a failure to recover the RNAPII pool is the principal reason for the lack of transcription recovery in CSB-deficient cells (Figure 7). Indeed, the mere introduction of non-degradable (K_1268_R) RPB1 in *CSB* KO cells restores transcription restart in these cells. This result has several important implications. First, most DNA lesions in genes are repaired 24 h after UV irradiation in the double *K*_*1268*_*R CSB KO* mutant; otherwise, RNAPII could not reach the end of the very long genes assayed for re-start here. This changes the way we think about TC-NER in human cells, and in CSB-deficient cells in particular. Indeed, direct measurements of strand-specific DNA lesion removal showed that CSB-deficient cell lines completely fail to preferentially repair lesions in the transcribed strand of active genes (Venema et al., 1990). However, transcription restart measured at the end of genes in the double *K*_*1268*_*R CSB KO* mutant indicates that these cells can repair the transcribed strand of genes. Needless to say, TC-NER absolutely requires RNAPII, which is dramatically depleted in CSB-deficient cell lines, but not in the *K*_*1268*_*R CSB KO* double mutant. It is thus possible that, as long as RNAPII is present, CSB is not absolutely required for the process of TC-NER in human cells. Interestingly in this regard, significant TC-NER still takes place in yeast when the gene encoding the CSB homolog Rad26 is deleted. Such *RAD26*-independent TC-NER is dependent on RNAPII itself as simultaneous deletion of both *RAD26* and *RPB9* (encoding a non-essential RNAPII subunit) completely abolishes TC-NER (Li and Smerdon, 2002). Our data thus suggest that the ability to perform Rad26/CSB-independent TC-NER may be conserved in evolution, but that this has been overlooked due to the dramatic effect of CSB on RNAPII stability in human cells. Despite being much slower than TC-NER, GG-NER might obviously contribute to the removal of transcription-blocking DNA damage as well. Indeed, the majority of UV-induced lesions will be repaired by this pathway, and most CPDs are removed by 24 h after UV irradiation in HEK293 cells (Figure S6I). Importantly, however, regardless of the repair pathway used by CSB-deficient cells to repair transcription-blocking DNA damage, the lack of transcription restart in these cells must still be caused by RNAPII depletion.

It is worth noting that, despite being able to restart transcription after DNA damage, double mutant *K*_*1268*_*R CSB* KO cells are still UV-sensitive. However, given that the single mutant cell types are both highly UV-sensitive, this is hardly surprising and might reflect either the slower TC-NER in these cells or point to a crucial role for correct transcriptional shutdown of short genes, such as growth-promoting IEGs during DNA damage.

### RNAPII Pools in Genome Instability Disorders and Beyond

Interestingly, the link between CSB, RPB1 stability, and transcription recovery upon DNA damage established here may also reconcile previously opposing views on the cause of Cockayne syndrome. The complex phenotype of this severe human disorder involves not only UV-sensitivity, but also progeroid features, and—most notably—severe defects in neuronal development, which are difficult to explain via DNA damage and repair defects (Brooks, 2013). Indeed, we and others have suggested that altered transcription programs in CS cells might explain several severe patient characteristics (Proietti-De-Santis et al., 2006, Vélez-Cruz and Egly, 2013, Wang et al., 2014, Wang et al., 2016). Interestingly, if we accept the possibility that CS cells generally fail to protect stalled RNAPII from degradation, then endogenous or exogenous sources of DNA damage, even in low doses, might aberrantly affect the RPB1 pool and thus affect transcription programs in CS patients. In this model, CS, and the CS-related features of certain Xeroderma pigmentosum patients, would indeed be caused by DNA damage, but not because of problems caused by the lesions themselves, but because they result in an abnormal regulation of the free RNAPII pool and thus perturb cell-specific transcription programs, causing neurodevelopmental abnormalities, et cetera.

Our data even open the intriguing possibility that global regulation of RPB1 stability, and the size of the RNAPII pool, might transcend the UV response, and contribute significantly to other genome instability disorders, and perhaps even to the regulation of cell-type-specific transcription programs in normal cells. Many chromosomal events that affect transcript elongation might thus disturb transcription programs by affecting the free RNAPII pool available for correctly regulated transcription. In this context, it is interesting that patients suffering from a number of genome instability disorders, including (for example) Fanconi anemia, Blooms syndrome, and Huntington’s disease (Jimenez-Sanchez et al., 2017, Rolig and McKinnon, 2000), have overlapping developmental disabilities. Likewise, some viruses inhibit host cell activation of innate immune responses by triggering a global depletion of RNAPII levels through RPB1 degradation (Akhrymuk et al., 2012, Verbruggen et al., 2011). We suggest that the effect on gene regulatory networks of a limited RNAPII pool has hitherto been incorrectly overlooked, and that even the change in the length of genes being actively transcribed in a certain cell type might affect the number of free RNAPII molecules available for new transcription (neuronal-specific genes are typically very long, for example). At the very least, the effect on RNAPII pool size and thereby altered transcription programs should be considered when the consequence of genome-destabilizing mutations or treatments is investigated.

## STAR★Methods

### Key Resources Table

**Table:** 

REAGENT or RESOURCE	SOURCE	IDENTIFIER
**Antibodies**		

RPB1 (total, N-terminal)	Cell Signaling	D8L4Y; RRID:AB_2687876
RPB1 (raised against S5-P, recognizes multiple forms)	Abcam	4H8; RRID:AB_304868
RPB1, serine 2 phosphorylated	kind gift from Dirk Eick	3E10
His-tag	Abcam	ab9108; RRID:AB_307016
CSB	Bethyl	A301-345A; RRID:AB_937849
XPD	abcam	ab150362; EPR9674
Vinculin	Sigma	V9131; RRID:AB_477629
Histone H3	Abcam	ab18521; RRID:AB_732917
CPSF73	Bethyl	A301-090A; RRID:AB_873009
FOS	Santa Cruz	sc-52; RRID:AB_2106783
FOSB	Cell Signaling	5G4; RRID:AB_2106903
ATF3	Cell Signaling	D2Y5W; RRID:AB_2799039
EGR1	Cell Signaling	15F7; RRID:AB_2097038
Tubulin (yeast)	Sigma	T6199; RRID:AB_477583
TAP-tag (yeast)	Thermo Fisher Scientific	CAB1001; RRID:AB_10709700
anti-mouse secondary antibody (HRP)	Santa Cruz	sc-516102; RRID:AB_2687626
anti-rabbit secondary antibody (HRP)	Jackson ImmunoResearch	711-035-152; RRID:AB_10015282
anti-rat secondary antibody (HRP)	Jackson ImmunoResearch	112-035-003; RRID:AB_2338128
Cyclobutane Pyrimidine Dimers (CPDs)	CosmoBio	TDM-2; RRID:AB_1962813

**Bacterial and Virus Strains**		

NEB® 5-alpha Competent *E. coli*	NEB	C2988J
One Shot BL21 Star (DE3)	Thermo Fisher Scientific	C601003

**Chemicals, Peptides, and Recombinant Proteins**		

Doxycycline	Clontech	8634-1
MG132	Cayman Chemical	10012628
N-Ethylmaleimide (NEM)	Sigma-Aldrich	E3876
4-thiouridine	Glentham Life Sciences	GN6085
4-thiouracil	Sigma-Aldrich	440736
DRB (5,6-dichloro-1-β-D-ribofuranosylbenzimidazole)	Sigma-Aldrich	D1916
MTSEA biotin-XX linker ((MTSEA Biotincapcap; 2-((6-((6-((biotinoyl)amino)hexanoyl)amino)hexanoyl)amino)ethylmethanethiosulfonate))	Biotium	BT90066
Dsk2 beads	Home-made; see Tufegdzic Vidakovic et al., 2019	N/A
HRP-conjugated streptavidin	Thermo Fisher Scientific	N100
Lipofectamine 3000	Thermo Fisher Scientific	L3000015
High glucose DMEM	Thermo Fisher Scientific	11965118
Tet-free FBS	Clontech	631106
Poly-lysine	Sigma-Aldrich	P7280
3-8% Tris-Acetate gels	BioRad	3450130
4-15% TGX gels (18wells/26/wells)	BioRad	56711084/5
Complete EDTA-free protease inhibitor cocktail	Sigma-Aldrich	05056489001
PhosSTOP	Sigma-Aldrich	04906837001
Nitrocellulose membrane	GE Healthcare Life Sciences	10600002
SuperSignal West Pico PLUS ECl reagent	Thermo Fisher Scientific	34577
Radiance Plus ECL	Azure Biosystems	AC2103
Protein G agarose beads	Thermo Fisher Scientific	20397
InstantBlue	Expedeon	ISB1L
Micro Bio-Spin P-30 Gel Columns	BioRad	7326223
iTaqUniversal SYBR® Green Supermix	BioRad	172-5124
Benzonase	MerckMillipore	70746-4
Lipofectamine 3000	Thermo Fisher Scientific	L3000015
AMPureXP beads	Beckman Coulter	A63881
TRIzol Reagent	Thermo Fisher Scientific	15596026

**Critical Commercial Assays**		

RNeasy kit	QIAGEN	74104
miRNeasy kit	QIAGEN	217004
RNA minElute clean-up kit	QIAGEN	74204
RNase-Free DNase Set	QIAGEN	79254
PureLink RNA Mini kit	Thermo Fisher Scientific	12183020
μMACS Streptavidin Kit	Miltenyi	130-074-101
Taqman Reverse Transcriptase Reagents	Thermo Fisher Scientific	N8080234
PTMScan Ubiquitin Remnant Motif (K-ε-GG) Kit	Cell Signaling Technology	#5562
TMT10plex Isobaric Label Reagent Set	Thermo Fisher Scientific	90110
KAPA RNA HyperPrep Kit	Kapabiosystems	KR1350
KAPA mRNA HyperPrep kit	Kapabiosystems	KK8581

**Deposited Data**		

Genome-wide data are available under GEO number GSE143542.	This manuscript	GEO: GSE143542

**Experimental Models: Cell Lines**		

Flp-In T-Rex HEK293 cells	Thermo Fisher Scientific	R78007
RPB1 K1268R knock-in clone E2 (in Flp-In T-Rex HEK293)	This manuscript	N/A
RPB1 K1268R knock-in clone D12 (in Flp-In T-Rex HEK293)	This manuscript	N/A
RPB1 K1350R knock-in clone F10 (in Flp-In T-Rex HEK293)	This manuscript	N/A
Switchover RPB1-His WT clone 9 (in Flp-In T-Rex HEK293)	This manuscript	N/A
Switchover RPB1-His WT clone 10 (in Flp-In T-Rex HEK293)	This manuscript	N/A
Switchover RPB1-His K1268R clone 3 (in Flp-In T-Rex HEK293)	This manuscript	N/A
Switchover RPB1-His K1268R clone 12 (in Flp-In T-Rex HEK293)	This manuscript	N/A
Switchover RPB1-His K619,627,642R clone 1 (in Flp-In T-Rex HEK293)	This manuscript	N/A
Switchover RPB1-His K710,719R clone 1 (in Flp-In T-Rex HEK293)	This manuscript	N/A
Switchover RPB1-His K751,758,761R clone 4 (in Flp-In T-Rex HEK293)	This manuscript	N/A
Switchover RPB1-His K1278R clone 8 (in Flp-In T-Rex HEK293)	This manuscript	N/A
Switchover RPB1-His WT CSB K.O. clone 1 (in Flp-In T-Rex HEK293)	This manuscript	N/A
Switchover RPB1-His K1268R CSB K.O. (double mutant) clone 2 (in Flp-In T-Rex HEK293)	This manuscript	N/A
CSB K.O. Flp-In T-Rex HEK293	This manuscript	N/A
CS1AN	Kind gift from Alan Lehman	N/A

**Experimental Models: Organisms/Strains**		

*S. cerevisiae* (strain BY4741, MATa, his3D1, leu2D0, met15D0, ura3D0)	Euroscarf	BY4741(Y00000)
*S. cerevisiae* BY4741 Rpo21K1246R HTP::URA	Milligan et al., 2017	N/A

**Oligonucleotides**		

All oligonucleotides are listed in Table S4	This manuscript	N/A

**Recombinant DNA**		

pDONR223	Kind gift from Simon Boulton	N/A
pENTR4 dual selection	Thermo Fisher Scientific	A10465
pFRT/TO	Kind gift from Markus Landthaler	N/A
pOG44	Thermo Fisher Scientific	V600520
pSpCas9(BB)-2A-GFP	Ran et al., 2013	Addgene #48138
pSpCas9n(BB)-2A-GFP	Ran et al., 2013	Addgene #48140
pFRT-TO-RPB1-His WT CRres si2,4R	This manuscript	Addgene #139404
pFRT-TO-RPB1-His K1268R CRres si2,4R	This manuscript	Addgene #139405
pFRT-TO-RPB1-His K1278R CRres si2,4R	This manuscript	Addgene #139406
pFRT-TO-RPB1-His K710,719R CRres si2,4R	This manuscript	Addgene #139407
pFRT-TO-RPB1-His K751,758,767R CRres si2,4R	This manuscript	Addgene #139408
pFRT-TO-RPB1-His CRres K619,627,642R si2,4R	This manuscript	Addgene #139409
pGEX3-Dsk2	Anindya et al., 2007	N/A
pGFP-CSB	van den Boom et al., 2004	N/A

**Software and Algorithms**		

Source code for the mathematical modeling of transcription	This manuscript	https://github.com/FrancisCrickInstitute/babs_uv_polymerase
MISO	Katz et al., 2010	https://www.genes.mit.edu/burgelab/miso/
MaxQuant version 1.3.05	Tyanova et al., 2016	https://www.maxquant.org
Perseus version 1.4.0.11	Tyanova et al., 2016	http://maxquant.net/perseus/
SAMtools	Li et al., 2009	http://www.htslib.org/
Bowtie version 2.2.3	Langmead and Salzberg, 2012	https://sourceforge.net/projects/bowtie-bio/files/bowtie2/2.2.3/
BEDtools	Quinlan and Hall, 2010	https://bedtools.readthedocs.io/en/latest/
PARalyzer	Corcoran et al., 2011	https://ohlerlab.mdc-berlin.de/software/PARalyzer_85/
Ngs.plot	Shen et al., 2014	https://github.com/shenlab-sinai/ngsplot
Cutadapt	Martin, 2011	https://cutadapt.readthedocs.io/en/stable/index.html
RSEM	Li and Dewey, 2011	https://github.com/deweylab/RSEM

### Lead Contact and Materials Availability

Further information and requests for resources and reagents should be directed to and will be fulfilled by the Lead Contact, Jesper Q. Svejstrup (jesper.svejstrup@crick.ac.uk). Plasmids were deposited with and will be distributed through the non-profit distributor Addgene (see Key Resources Table for plasmid codes). Cell lines generated in this study are available from the Lead Contact without restriction.

### Experimental Model and Subject Details

#### Cell lines and culture conditions

Flp-In T-REx HEK293 (Thermo Fisher Scientific) cell lines were cultured in standard Dulbeco’s modified Eagle’s medium (DMEM) supplemented with 10% FBS, 100 U/mL penicillin, 100 μg/mL streptomycin, 2 mM L-glutamine, and in the case of stable cell lines, Hygromycin B (0.1 mg/ml) and Blasticidine (0.015 mg/ml). Stable cell lines expressing siRNA-resistant, doxycycline-inducible His-tagged RPB1 (WT or various K→R mutants) were generated using the Flp-In system, individual colonies were selected by hygromycin, and monoclonal cell lines were isolated. *K_1268_R* and *K_1350_R* knock-in cell lines were created in Flp-In T-REx HEK293 background using HDR-CRISPR editing (Richardson et al., 2016) and FACS sorted as single cells to obtain monoclonal cell lines. siRNA transfections were performed with Lipofectamine RNAiMax (Thermo Fisher) according to manufacturer instructions, and 40 nM final siRNA concentration was used. UVC-irradiation was performed either in a UV-crosslinker (Stratagene) or a custom-built UV-conveyor belt (Tufegdzic Vidakovic et al., 2019), and the given dose was monitored with a UV-meter.

### Method Details

#### Generation of stable cell lines and CRISPR knock-in cells

The RPB1 *(POLR2A*) coding region with 6x His tag at the C terminus was obtained by gene synthesis from GeneArt (ThermoFisher), carrying several synonymous mutations providing resistance to targeting by two guide RNAs (“CRISPR res”) and restriction enzymes potentially used for RFPL analysis (CviQ, Hpy188I and Mwo). Site directed mutagenesis was further performed to introduce synonymous mutations providing resistance to two siRNAs targeting RPB1 (D-011186-03-0020 and D-011186-05-0020, Dharmacon) and resistance to XhoI restriction endonuclease within the RPB1 open reading frame, to enable further cloning. The RPB1-His coding construct was cloned into pFRT-TO plasmid, and named pFRT-TO-wtRPB1-His-siR. This construct was then subjected to site directed mutagenesis to introduce lysine-to-arginine (K → R) mutations at desired ubiquitylation positions (mutagenesis primers provided in the Key Resources Table). The above plasmids were used to generate Flp-In T-REx HEK293 stable cell lines with doxycycline-inducible WT or K → R mutated RPB1-His. Briefly, Flp-In T-REx HEK293 cell lines were co-transfected with a 9:1 ratio of pOG44 Flp-recombinase expression vector (Thermo Fisher Scientific, V600520) and pFRT-TO-(wt / K → R)RPB1-His-siR constructs using Lipofectamine 2000 (Thermo Fisher Scientific, 11668019), according to the manufacturer’s instructions. 24 h after transfection, single cells were seeded in 15 cm dishes and after another 24 h the cell culture media was supplemented with 100 μg/mL hygromycin and 15 μg/mL blasticidin. Selection was performed for 2 weeks and single colonies were isolated and propagated. Expression of RPB1-His was induced by the addition of doxycycline (Clontech, 8634-1, 500 ng/mL final concentration) and verified by western blot using antibodies against N-terminal part of RPB1 (D8L4Y) and His-tag (ab9108).

*K*_*1268*_*R* and *K*_*1350*_*R* knock-in cells were generated by editing the endogenous RPB1 (*POLR2A*) locus using homology-directed repair CRISPR technology. Briefly, Flp-In T-REx HEK293 cells were co-nucleofected with asymmetric single stranded donor DNAs (Key Resources Table) and a pSpCas9(BB)-2A-GFP vector encoding GFP-tagged Cas9 nuclease and the corresponding guide RNA (Key Resources Table). Two days after nucleofection, single GFP+ cells were sorted by FACS into 96-well plates. Upon formation of mono-clonal colonies, individual colonies were screened for the successful editing of the endogenous RPB1 (*POLR2A*) locus by genomic DNA extraction using Quick Extract kit (QE0905T), followed by PCR amplification of the corresponding gene regions. PCR reactions were cleaned up by treatment with ExoSAP-IT PCR Product Cleanup Reagent (Thermo Fisher Scientific) and subjected to Sanger sequencing. The data were analyzed manually, using Serial cloner and ApE software.

#### Generation of CSB KO cell lines

CRISPR-Cas9-nickase-mediated genome editing of Flp-In T-REx HEK293 cell lines was performed as previously described (Ran et al., 2013). The oligonucleotides encoding gRNAs for targeting exon 2 of the coding region of *ERCC6* are listed in the Key Resources Table. Briefly, the forward and reverse strand oligonucleotides were annealed and ligated into pSpCas9n(BB)-2A-GFP linearized with BbsI, and plasmids were sequenced after cloning and transformation. To generate knockouts, cells were co-transfected with the two pSpCas9n(BB)-2A-GFP plasmids containing nickase-gRNA pairs 1 and 2 using Lipofectamine 3000 (Thermo Fisher Scientific) according to the manufacturer’s instructions. 48 h after transfection, high GFP-positive cells were sorted clonally by FACS into 96-well plates and cultivated until colonies were obtained. Clones were tested for the presence of ERCC6 by western blotting and clones with complete absence of ERCC6 were saved.

#### UV irradiation and chemical treatments of human cells

UV irradiation was performed as described in detail previously (Tufegdzic Vidakovic et al., 2019). Either Stratalinker (Stratagene) or a custom-made UV conveyor belt were used. In every experiment, the exact given doses were monitored using a UV-meter (Progen Scientific). For growth analysis using Incucyte (Sartorius), the cells were treated with 4-NQO (Sigma) for 1 h and cisplatin (Sigma) for 1 h. For the DRB stability experiment, the cells were either untreated or UV-irradiated, and media containing 100 μM DRB was immediately added to the cells.

#### Detection of ubiquitylated RPB1

GST-Dsk2 pulldown of ubiquitylated proteins in yeast and human cells has recently been described in detail (Tufegdzic Vidakovic et al., 2019). To prepare Dsk2 beads, One Shot BL21(DE3) Star competent bacterial cells were transfected with with pGEX3-Dsk2 plasmid according to the manufacturer’s instructions and plated on ampicillin selection plates. After overnight incubation at 37°C a colony was picked and inoculated into 20 mL of LB containing 100 μg/ml ampicillin (LB_amp_), and grown at 37°C overnight with shaking (pre-inoculum culture). The following day, 300 mL of LB_amp_ were inoculated with 5 mL of the pre-inoculum in 2L Erlenmeyer flask, and grown at 37°C with shaking at 200 rpm. Expression of GST-Dsk2 was induced with 1mM IPTG when bacterial growth reached the absorbance at 600 nm (OD600) of 0.8. The induced culture was grown for 4 h at 30°C, with shaking at 200 rpm. Bacteria were then centrifuged and the pellets were snap frozen in liquid nitrogen, and stored at −80°C until further processing. The pellets from 300 mL culture were defrosted quickly at room temperature, transfered to ice and resuspended in 90 mL cold PBSA containing protease inhibitors. Suspensions were then sonicated with a tip probe sonicator (Branson Digital Sonifier 250) at 33% output, with 15 s ON, 30 s OFF pulses, for a total ON pulse duration of 10 min. Triton X-100 was added to the suspension to a final concentration of 0.5% and mixed gently, and lysates were then incubated on ice for 30 min and centrifuged at 12,000 rpm for 10 min at 4°C to remove debris. Supernatants were saved in fresh tubes and then added to the prewashed (2 washes in PBSA) glutathione Sepharose beads. 1 mL of original bead suspension (0.5 mL of packed beads) were used per 30 mL of cleared lysate. DTT was added to a final concentration of 2 mM, and suspensions were incubated in the cold room for at least 4 h or overnight, with gentle rotation. The following day, the beads were span down at 500 g for 5 min at 4°C, and the pellet (GST-Dsk2 beads) was washed twice with ice-cold PBSA, 0.1% Triton X-100, containing protease inhibitors, and then washed once more with PBSA without Triton X-100, but containing protease inhibitors. 30 mL of PBSA containing protease inhibitors and 0.02% sodium azide were added to the prepared Dsk2 beads and stored at 4°C.

Human cell lysates were prepared by scraping the cells in PBS, spinning down and removing the supernatant. Cell pellet was resuspended in TENT buffer (50 mM Tris-HCl pH 7.4, 2 mM EDTA, 150 mM NaCl, 1% Triton X-100) containing protease inhibitors, phosphatase inhibitors and 2 mM of N-ethylmaleimide [NEM]. Note that the NEM stock solution (200 mM in ethanol) should be made up fresh every time. After incubation on ice for 10 min, the samples were sonicated in a 4°C water bath sonicator (Bioruptor) at high power, with 30 s ON and 30 s OFF pulses, for a total duration of 7 min, then centrifuged at maximum speed (14,000 rpm) for 5 min to remove debris. Yeast extracts were prepared as described in “*Yeast cell growth and 4-NQO treatment”* section below.

Prepared Dsk2 beads were pre-washed in TENT buffer containing protease inhibitors, phosphatase inhibitors and 2 mM NEM. 0.5 mL of GST-Dsk2 bead suspension (equivalent to 25 μL packed beads) were used to enrich ubiquitylated proteins from 1 mg of whole cell protein extract. Samples were rotated on a turning wheel/rotator (low to moderate speed) in the cold room for several hours to overnight (human cells) or two hours (yeast cells). For pulldowns from human cell lystaes, the beads were then washed twice with 1 mL of TENT buffer containing protease inhibitors, phosphatase inhibitors and 2 mM NEM, and then once with 1 mL of PBS containing protease inhibitors, phosphatase inhibitors and 2 mM NEM. For pulldowns from yeast extracts, the beads were washed once in the extract buffer, followed by two washes in the extract buffer with 300 mM potassium acetate, before a final wash in extract buffer. The samples were then centrifuged at 500 g for 5 min at 4°C, all liquid was removed, and 40 μL of Laemmli buffer containing DTT or β-mercaptoethanol were added to the beads, the samples were mixed by brief vortexing, and boiled at 96-98°C for 5 min. Samples were span down and supernatants were saved and analyzed by Western Blot (see section “*Western Blot*”). For more detailed protocols please refer to Tufegdzic Vidakovic et al. (2019).

#### Immunoprecipitation of RPB1

RPB1 immunoprecipitations (IPs) were performed from chromatin fractions. Two p15 dishes per condition were used. The cells were UV-irradiated as outlined in detail previously (Tufegdzic Vidakovic et al., 2019). The cells were scraped in PBS, washed once with PBS and span down, and the pellets were then snap-frozen in liquid nitrogen. For chromatin extraction, the frozen pellets were quickly defrosted at room temperature and transferred to ice. 2 pellet volumes of hypotonic buffer were added (10 mM HEPES-KOH pH 7.5, 10 mM KCl, 1.5 mM MgCl_2_, with addition of 20 mM freshly made N-ethylmaleimide [NEM], protease and phosphatase inhibitors) and the cells were incubated on ice for 20 min. To release cytosol, 20 strokes with a loose pestle were applied to the samples in a dounce-homogenizer. To pellet nuclei, the samples were centrifuged at 1,000 g at 4°C for 20 min. Supernatant (cytosolic fraction) was removed, and pellets were resuspended by pipetting in 2 pellet volumes of nucleoplasmic extraction buffer (20 mM HEPES-KOH pH 7.5, 1.5 mM MgCl_2_, 10% glycerol, 150 mM potassium acetate, 0.05% NP-40, with addition of protease and phosphatase inhibitors), and incubated on a turning wheel in the cold room for 20 min. To separate nucleoplasm from chromatin, the samples were centrifuged at 20,000 g at 4°C for 20 min. The supernatant (nucleoplasmic fraction) was removed and chromatin pellets were resuspended in 1 pellet volume of chromatin digestion buffer (125 U/ml benzonase in 20 mM HEPES-KOH pH 7.5, 1.5 mM MgCl_2_, 10% glycerol, 150 mM NaCl, 0.05% NP-40, with addition of protease and phosphatase inhibitors). The samples were incubated on ice for at least 30 min, then centrifuged at 20,000 g at 4°C for 20 min. Supernatant (first chromatin fraction) was saved in a new Eppendorf tube. The remaining chromatin pellet was resuspended in 1 pellet volume of chromatin-2 buffer (20 mM HEPES-KOH pH 7.5, 1.5 mM MgCl_2_, 10% glycerol, 3 mM EDTA, 500 mM NaCl, 0.05% NP-40, with addition of protease and phosphatase inhibitors) and incubated on a turning wheel in the cold room for 20 min. 2.3 pellet volumes of dilution buffer (20 mM HEPES-KOH pH 7.5, 1.5 mM MgCl_2_, 10% glycerol, 3 mM EDTA, 0.05% NP-40, with addition of protease and phosphatase inhibitors) were added to each sample, and samples were then centrifuged at 20,000 g at 4°C for 15 min. Supernatant (second chromatin fraction) was saved and pooled with the first chromatin fraction.

1 mg of chromatin fraction were used per IP (all samples were adjusted to the same volume, typically 800-950 μl). For immunoprecipitation of RPB1 with 4H8 antibody, 100 μL of packed Protein G agarose beads (Thermo Fisher Scientific, 20397) per sample were prepared, by washing twice in 0.05% Tween in PBS (PBST) and then coupling to 30 μL of 4H8 antibody (1 mg/ml stock) per sample, for 1 h on a turning wheel at room temperature. The beads were then washed twice in PBST and once in IP buffer (20 mM HEPES-KOH pH 7.5, 1.5 mM MgCl_2_, 10% glycerol, 150 mM NaCl, 0.05% NP-40) containing phosphatase and protease inhibitors. The beads were then resuspended in IP buffer containing phosphatase and protease inhibitors and added to the samples, to yield a 1 mL total reaction volume. The samples were incubated on a turning wheel in the cold room for 3 hours, and then centrifuged at 500 g, at 4°C for 3 min. Supernatant (unbound fraction) was removed and saved, and the beads were washed 3 times in IP buffer. To elute immunoprecipitated proteins, 100 μL of 2X Laemli buffer were added to the beads, the beads were briefly vortexed and boiled at 98°C for 5 min. The beads were centrifuged at maximum speed for 2 min and the supernatant (elution) was carefully removed and transferred to a new tube.

Proteins were resolved on 4%–15% TGX gels and transferred to nitrocellulose membranes using wet transfer at 0.5 mA constant current for 1 h, in a transfer buffer without SDS or alcohols (25 mM Tris, 192 mM glycine). After Ponceau S staining, the membranes were blocked in 5% milk in PBST for 1 h at room temperature. Primary antibodies were applied at 4°C overnight, in 5% milk-PBST. For the list of antibodies used refer to the Key Resources Table.

#### Western blot

For whole cell extracts, cells pellets were resuspended in protein lysis buffer (20 mM Tris-HCl pH 7.5, 250 mM NaCl, 1 mM EDTA, 0.5% (v/v) NP-40, 10% glycerol) and briefly sonicated using Bioruptor water bath sonicator (Diagenode) at a high setting, with 30 s ON / 30 s OFF, for the total duration of 7 min. To clear the lysates, the samples were centrifuged at 4°C at maximum speed for 7 minutes. Proteins were separated on 3%–8% Tris-Acetate (BioRad, 3450130) or 4%–15% TGX gels (BioRad, 56711084/5) and transferred to nitrocellulose membranes (GE Healthcare Life Sciences, 10600002). Membranes were blocked in 5% (w/v) skimmed milk in PBST (PBS, 0.1% (v/v) Tween20) for 1 h at room temperature and incubated with primary antibody (in 5% (w/v) skimmed milk in PBST) overnight at 4°C. Primary antibodies are listed in Key Resources Table. Antibody against Vinculin served as a loading control. Membranes were washed several times in PBST, incubated with HRP-conjugated secondary antibody (Key Resources Table) in 5% (w/v) skimmed milk in PBST and visualized using SuperSignal West Pico PLUS (Thermo Fisher Scientific, 34577) or Radiance plus Chemiluminescent Substrate ECL reagent (Azurebiosystems).

#### Yeast cell growth and 4-NQO treatment

*Saccharomyces* cerevisiae strains BY4741Rpo21HTP::URA3 and BY4741 Rpo21K1246R HTP::URA (Milligan et al., 2017) were grown and manipulated using standard techniques (Sherman, 1991). Damage-induced Rpb1 degradation and ubiquitylation in *Saccharomyces cerevisiae* was investigated after exposure to 4-NQO using early logarithmic cells (Tufegdzic Vidakovic et al., 2019, Wilson et al., 2013b). 4-NQO was used at a final concentration of 10 μg/ml (10 mg/ml stock solution in DMSO) and incubated with cells for the indicated times. For Rpb1 degradation, whole cell extracts were prepared by alkaline (denaturing) extraction (Kushnirov, 2000). In order to visualize ubiquitylated yeast Rpb1, the total ubiquitylated protein pool was isolated from whole cell extracts prepared by bead-beating in lysis buffer (150 mM Tris-acetate pH 7.4,100 mM potassium acetate, 1 mM EDTA, 0.1% Triton X-100, 10% glycerol, 2 mM NEM, 10 μM MG132 and protease inhibitors).

To measure UV sensitivity, cells were grown to an OD of 0.5 in YPDA and diluted to OD 0.1. The cells were then serially diluted five-fold and spotted on to YPDA agar plates. The plates were then UV-irradiated before incubation for 3 days at 30°C.

#### Human cell colony forming assays and growth assays

Switchover stable cell lines were seeded in 6 well plates in the presence of 10 ng/ml doxycycline (Dox). The following day, the cells were transfected using Lipofectamine RNAiMax (Thermo Fisher Scientific) with a pool of two individual siRNAs (D-011186-03-0020 and D-011186-05-0020, Dharmacon) against POLR2A (RPB1 encoding transcript), at a total 40 nM final concentration (20 nM each siRNA). The following day, single cells were seeded in 6-well plates in the presence of 500 ng/ml Dox (500 cells/well for untreated cells, 1000-4000 cells/well for UV). Parental Flp-In T-REx HEK293, *K*_*1268*_*R* CRISPR knock-in and *CSB* knock-out cells were seeded in regular media, at the same densities as the switchover cell lines. Two days after seeding, the cells were UV irradiated. Colonies were fixed by 4% (v/v) formaldehyde 7-10 days after seeding and stained with a 0.1% (w/v) crystal violet solution. Quantification was performed by counting colonies from three biological replicates. For growth assays, 5,000 – 20,000 cells were seeded per well in poly-lysine coated 96-well plates, and UV irradiated or treated with indicated chemicals two days later. Growth was monitored and recorded every 3-4 h using Incucyte (Essenbioscience), and the growth in each well was normalized to the starting cell density in that well (time point = 0). Data from 2-3 biological replicate series, each with 3-6 technical replicate wells were used for plotting.

#### RT-qPCR

For RT-qPCR, total RNA was extracted using miRNeasy kit (QIAGEN, 217004) or RNeasy kit (QIAGEN, 74104), following the instructions of the manufacturer including an on-column DNase treatment (QIAGEN, 79254). 1 μg of RNA per sample were first denatured at 65°C for 5 min and snap cooled on ice, then used for reverse transcription with TaqMan Reverse Transcription Reagents (Thermo Fisher Scientific, N8080234). For detection of nascent RNA, reverse transcription was performed with random hexamers, and for mature RNA with oligo dT primers. 3 μL of cDNA diluted with water (typically 1:3 – 1:5) were used per well for qPCR with iTaq Universal SYBR Green Supermix (BioRad, 172-5124), with 30 cycles of 15 s denaturation at 94°C, 15 s annealing at 58°C, and 20 s extensions at 72°C. Primers amplifying mature GAPDH were used as normalization control. Primer sequences are listed in the Key Resources Table. Data were analyzed using Excel and plotted in GraphPad Prism. A minimum of three biological replicates were always analyzed, each in technical triplicate.

#### Laser-stripe micro-irradiation

2 × 10^6^ cells were plated in MatTek glass bottom dishes (35 mm, No. 2 14mm diameter glass) phenol-red free DMEM (GIBCO, Invitrogen) supplemented with 5% Glutamax, 10% Fetal Bovine Serum (GIBCO, Life Technologies), 2 mM L-glutamine, 1% Pen-Strep, and 5 μg/mL blasticidin. Cells were grown in a 37°C incubator at 5% CO2. Cells were transfected with 100 ng plasmid encoding GFP-CSB (van den Boom et al., 2004) using FuGENE HD. 24 hours later, culture medium was replaced with phenol-red free medium containing the same additives plus 1 μg/ml Hoechst 33258 for 30 minutes to label nuclei and sensitize cells to laser micro irradiation.

Laser-micro irradiation was performed by subjecting cell nuclei to micro-irradiation with a 405 nm laser in a 200 × 3 pixel (34 × 0.51 μm) stripe (Weems et al., 2019). Micro-irradiation was performed with a 405 nm laser. Cells were exposed to 500-700 μW for approximately 3 s (40 iterations) at 100% laser power. Normal cell and nuclear morphology were preserved over the timescale of the experiment. GFP-CSB was excited with the 488 nm laser and imaged through a 500-550 emission filter. 488 nm laser power and exposure time were adjusted to maximize image quality and minimize photobleaching; absence of significant photobleaching was confirmed by observing unperturbed cells in the acquisition field of view. Micro-irradiation and imaging were performed on a Perkin Elmer UltraVIEW VoX spinning disk microscope, which included a Yokagawa CSU-X11 spinning disk, an ORCA-R2 camera (Hamamatsu), and a Perkin Elmer PhotoKinesis accessory. The microscope base was a Carl Zeiss Axiovert 200M equipped with a 40x 1.3 NA Plan-Apochromat objective and a 37°C, 5% CO_2_ incubator (Solent Scientific).

Quantitation and values for normalized recruitment after micro irradiation (Rt) were calculated using the equation R (t) = [I (t)/T (t)] / [I (0)/T (0)]. I (0) and T (0) are the average fluorescence intensities of the micro irradiated and total nuclear region, respectively, averaged over the pre-irradiation time period. (I (t) is the fluorescence intensity of the micro irradiated stripe as a function of time and was measured as the average intensity of a manually selected region corresponding to the visible bleached region immediately after micro irradiation. T (t) (total nuclear fluorescence intensity) was measured in the same way selecting the nuclear boundary. Original data for these experiments can be accessed from the Stowers Original Data Repository at https://www.stowers.org/research/publications/LIBPB-1491.

#### Immunofluorescence for detection of cyclobutane pyrimidine dimers (CPDs)

HEK293 cells were grown in poly-Lysine coated coverslips in a 12-well plate at a density of 200,000 cells per well. The next day, cells were irradiated with 15 J/m^2^ of UVC and fixed at different times after irradiation in 4% formaldehyde (15 minutes at RT). After washing twice in PBS, cells were permeabilized with 0.5% of Triton X-100 for 5 minutes at RT and washed twice with PBS. To allow for CPD antibody recognition, denaturation of the samples was achieved by treating with 2N HCl for 30 minutes at RT followed by 2 washes in PBS. For blocking, coverslips were treated with 0.1% Tween and 1% BSA in PBS for 1 hour at RT and washed twice with PBS. Primary antibody (mouse monoclonal, cosmobio CAC-NM-DND-001, clone TDM-2) was added in a 1:1500 dilution for 1 h at RT and washed 3 times with PBS. Secondary antibody (Alexa Goat anti Mouse 594, thermo A-11005) was added to the samples at 1:1000 and incubated for 1 hour at RT. Coverslips were washed 3 times in PBS and mounted onto slides using VECTASHIELD Antifade Mounting Medium containing DAPI (Vector Laboratories, H-1200) and visualized using an upright 780 confocal Zeiss microscope.

#### Quantitative multiplexed profiling of ubiquitylation

##### Cell lysis, trypsin digestion and immunoprecipitation of diGly-Containing Peptides

Cells were lysed in 9 M urea, 20mM HEPES (pH = 7.8), supplemented with 100 units/ml of benzonase and sonicated to reduce viscosity (3 mm probe, 50% amplitude, 3 × 15 s bursts, on ice). Total of 10 mg of protein per sample were used as estimated by Bradford protein assay. Lysates were reduced with 10 mM dithiothreitol (DTT) for 30 min at room temperature, followed by alkylation with 20 mM chloroacetamide for 30 min at room temperature in the dark. Lysates were digested initially with LysC (Promega) for 2 hours at 37°C. The lysates were then diluted with 20 mM HEPES, 5% acetonitrile to a final urea concentration of less than 2 M. The samples were digested 1:100 enzyme to protein ratio (w/w) with trypsin (Promega) overnight at 37°C. The next day, two additional aliquots of trypsin were added and incubated at 37°C four hours each. After the digestion the samples were acidified with TFA (Thermo Fisher Scientific) to final concentration of 1% (v/v). All insoluble material was removed by centrifugation and the supernatant was desalted with C18 SepPak solid-phase extraction cartridges (SPE) (Waters) and lyophilized for 2 days.

Peptides containing the diGly remnant were enriched using K-ε-GG affinity resin (Cell Signaling Technology) according to the manufacturer’s instructions. Briefly, digests were reconstituted in 1.4 mL of immunoaffinity purification (IAP) buffer as supplied by the manufacturer. One aliquot (∼40 μL packed bead volume) was washed four times with PBS and split into ten. Incubation of sample and beads was performed with gentle end-over-end rotation at 4°C for 2 hours followed by a 30 s 2000 × *g* spin to pellet the beads. The antibody beads were washed twice with ice-cold IAP buffer followed by three washes with ice-cold water. DiGly peptides were eluted from the beads with the addition of 2 × 50 μL of 0.15% TFA and allowed to stand at room temperature for 5 min. After a 30 s 2000 × *g* spin, the supernatant containing the eluted diGly peptides was carefully removed and lyophilized.

##### Tandem Mass Tag (TMT) Labeling and Sample Fractionation

Samples enriched for peptides containing the diGly remnant were resuspended in 20 μL of 20 mM HEPES (pH 8.5). Isobaric labeling of the peptides was performed using the 10-plex tandem mass tag (TMT) reagents (Thermo Fisher Scientific). TMT reagents (0.8 mg) were dissolved in 40 μL anhydrous acetonitrile (ACN) of which 4 μL were added to the peptides along with 8 μL of ACN (30% (v/v) ACN final concentration). After 1 hr at room temperature, the reaction was quenched with 2 μL of 5% hydroxylamine. Labeled peptides were combined, acidified with FA (pH ∼2) and dried via vacuum centrifugation.

For fractionation the high pH reversed-phase peptide fractionation kit (Thermo Fisher Scientific) was used. The peptides were resuspended in 0.1% TFA and separated into seven fractions with increasing concentration of acetonitrile (10%, 15%, 17.5%, 20%, 25% and 50%) according to manufacturer’s instructions.

##### LC-MS/MS analysis

Peptide mixtures from TMT 10-plex labeled samples were chromatographically resolved on an EASY-spray PepMap RSLC C18 column (2μm, 100Å, 75μm X 50cm ID) using an Ultimate 3000 RSLCnano system (Thermo Fisher Scientific) over a 180 min gradient at 40°C. The peptides were separated using linear gradient of 2%–35% solvent B over 153 min. The column was washed with 95% B for 10 min and equilibrated with 2% B for the rest of the acquisition. Solvent A was 0.1% FA and 5% DMSO in HPLC-grade water, and solvent B was 0.1% FA and 5% DMSO in 80% acetonitrile and the flow rate was 300 nL/min. All spectra were acquired using an Orbitrap Fusion Lumos Tribrid mass spectrometer (Thermo Scientific). Xcalibur 2.0 software (Thermo Scientific) was used to control data acquisition. The instrument was operated in data dependent acquisition mode with top scan speed set at 3 s. MS1 spectra were acquired in the Orbitrap at a resolution of 120 000 and an ion target of 4E^5^. The MS2 precursors were isolated using the quadrupole (0.7 Th window) and analyzed in the Orbitrap at 60 000 resolution, with an AGC target of 1E^5^ and a max injection time of 105 ms. Precursors were fragmented by HCD at a normalized collision energy (NCE) of 38%.

For the SPS-MS3 method precursors were fragmented by CID at a normalized collision energy (NCE) of 35%. Following acquisition of each MS2 spectrum, a synchronous-precursor-selection (SPS) MS3 scan was collected on the top 10 most intense ions in the MS2 spectrum when the diagnostic 344.2137 ion (GlyGly-TMT) was detected. SPS-MS3 precursors were fragmented by high energy collision-induced dissociation (HCD) and analyzed using the Orbitrap (NCE = 65%, AGC = 1E^5^, maximum injection time = 500 ms, and resolution = 60 000).

##### Database search for peptide and protein identification

All raw data files were analyzed using the MaxQuant computational platform (*version* 1.5.2.8). MS2 spectra were searched against UniProt database specifying *Homo sapiens* (Human) taxonomy (Proteome ID: UP000005640; Organism ID: 9606, number of entries: 21039). A list of 247 common laboratory contaminants provided by MaxQuant was also added to the database. Searches were performed using “Reporter ion MS2” or “Reporter ion MS3” with “10-plex TMT” as isobaric labels. Carbamidomethylation of cysteines was specified as fixed modification, oxidized methionines, N-terminal protein acetylation and di-glycine-lysine were searched as variable modifications. The datasets were filtered on posterior error probability to achieve 1% false discovery rate on protein, peptide and site level.

#### 4SU slot blot

4SU labeling, RNA isolation and biotinylation were performed as for TT-seq (without RNA fragmentation). 400 ng of RNA per sample were immobilzed on Hybond nylon membrane (GE Healthcare, RPN203B). RNA was crosslinked to the membrane by two pulses of 2000 μJ UVC exposure. The membrane was blocked by incubation in blocking solution (10% SDS, 1 mM EDTA in PBS) for 20 min at room temperature, followed by incubation with 1:50,000 dilution of 1 mg/mL streptavidin-horseradish peroxidase (Pierce) in blocking solution, in dark at room temperature, for 15 min. The membrane was washed six times in PBS containing decreasing concentrations of SDS (10%, 1%, and 0.1% SDS, applied twice each) for 10 min. The signal of biotin-bound HRP was visualized by 1:5 diluted ECL detection reagent (SuperSignal West Pico PLUS, Thermo Fisher Scientific, 34577). As a loading control, the membrane was stained with methylene blue solution (0.5 M sodium acetate, 0.5% methylene blue) for 10 min and de-stained in water over night.

#### TT-seq (nascent RNA-seq)

TT-seq was performed essentially as described (Gregersen et al., 2019, Gregersen et al., 2020). Biological triplicates were generated for each condition. Each switchover stable cell line was seeded in four 6 cm dishes: 2 × 6 cm dishes in the presence of 10 ng/ml Dox and 2 × 6 cm dishes without Dox. The following day, the cells were transfected using Lipofectamine RNAiMax (Thermo Fisher Scientific), those in Dox-containing media with a pool of two individual siRNAs (D-011186-03-0020 and D-011186-05-0020, Dharmacon) against POLR2A (RPB1 encoding transcript), and those in media without Dox with non-targeting control siRNA (D-001206-14-20), all at a total 40 nM final concentration. The following day, cells from 2 × 6 cm dishes were re-seeded into 6 × 6 cm dishes in the presence (siRPB1) or absence (siControl) of 500 ng/ml Dox. Two days after re-seeding, the cells were UV-irradiated with 20 J/m^2^ UVC and *in vivo* labeling of nascent RNA was achieved with 1 mM 4SU (Glentham Life Sciences, GN6085) pulse for exactly 15 min (for 45 min time point samples, 4SU was added 30 min after the UV treatment, and for 3 h time point samples, 4SU was added 2 h and 45 min after the UV treatment). Labeling was stopped by TRIzol (Thermo Fisher Scientific, 15596026) and RNA extracted accordingly to the manufacturer’s instructions.

As a control for equal sample preparation, *S. cerevisiae* (strain BY4741, MATa, his3D1, leu2D0, met15D0, ura3D0) 4-thiouracil (4TU)-labeled RNA was spiked in to each sample. *S. cerevisiae* were grown in YPD medium overnight, diluted to an OD600 of 0.1, and grown to mid-log phase (OD600 of 0.8) and labeled with 5 mM 4TU (Sigma-Aldrich, 440736) for 6 min. Total yeast RNA was extracted using the PureLink RNA Mini kit (Thermo Fisher Scientific, 12183020) following the enzymatic protocol.

For purification of 4SU labeled RNA, 100 μg human 4SU labeled RNA was spiked-in with 1/100 of 4TU-labeled *S. cerevisiae* RNA. The 101 μg RNA (in a total volume of 100 uL) was fragmented by addition of 20 μL 1 M NaOH and left on ice for 20 min. Fragmentation was stopped by addition of 80 μL 1 M Tris pH 6.8 and cleaned up twice with Micro Bio-Spin P-30 Gel Columns (BioRad, 7326223) according to the manufacturer’s instructions. Biotinylation of 4SU- and 4TU- residues was carried out in a total volume of 250 μl, containing 10 mM Tris-HCl pH 7.4, 1 mM EDTA and 5 mg MTSEA biotin-XX linker (Biotium, BT90066) for 30 min at room temperature in the dark. RNA was then purified by phenol:chloroform extraction, denatured by 10 min incubation at 65°C and added to 200 μL μMACS Streptavidin MicroBeads (Milentyl, 130-074-101). RNA was incubated with beads for 15 min at room temperature and beads applied to a μColumn in the magnetic field of a μMACS magnetic separator. Beads were washed twice with pull-out wash buffer (100 mM Tris-HCl, pH 7.4, 10 mM EDTA, 1 M NaCl and 0.1% Tween20). Biotinylated RNA was eluted twice by addition of 100 mM DTT and cleaned up using the RNeasy MinElute kit (QIAGEN, 74204) using 1050 μL 100% ethanol per 200 μL reaction after addition of 750 μL RLT buffer to precipitate RNA < 200 nt.

Libraries for RNA sequencing were prepared using the KAPA RNA HyperPrep Kit (KR1350) with modifications. 75 ng of RNA per sample were mixed with FPE Buffer, but fragmentation procedure was omitted and RNA was instead denatured at 65°C for 5 min. The rest of the procedure was performed as recommended by the manufacturer, with the exception of SPRI bead purifications: after adaptor ligation, 0.95x and 1x SPRI bead-to-sample volume ratios were used (instead of two rounds of SPRI purification with 0.63x volume ratios). This was done to retain smaller (150-300 bp) cDNA fragments in the library which would otherwise be lost in purification. Libraries were amplified using 6 cycles of PCR. The libraries were then sequenced with single end 75 bp reads on the HiSeq 2500, with 50,000 reads per sample.

#### polyA+ mRNA-seq

Biological triplicates were generated for each condition. Switchover stable cell lines were seeded in 6-well plates in the presence of 10 ng/ml Dox. Transfection with a pool of two siRPB1 siRNAs and re-seeding timeline were the same as for TT-seq and colony forming assays. Two days after re-seeding into 6-well plates, the cells were UV-irradiated with 20 J/m^2^ UVC and collected 3 h, 8 h, 24 h and 48 h later. RNA was extracted using miRNeasy kit (QIAGEN) and 2 μg of RNA was used per condition for polyA+ mRNA library preparation. Libraries were generated using KAPA mRNA HyperPrep kit (KK8581), and sequenced with 75 bp paired end reads on HiSeq2500, with 40,000 reads per sample.

### Quantification and Statistical Analysis

#### Analysis of RT-qPCR data

Biological triplicates (each in technical triplicate) were assayed for each condition, and the data were analyzed using multiple t tests with Holm-Sidak correction. Analysis details are also included in Figure legends.

#### Mathematical modeling and *in silico* simulations

To model polymerase dynamics numerically, we implemented a process-oriented simulator, continuous in time and location along gene, but discrete in events. Thus, a set of biological events (see Table S3 for a complete list, but for example repair, transcription completion, polymerase collision with a stalled polymerase) each have a time associated with them. Between events, transcription proceeds in a continuous manner, but when an event occurs an abrupt update is made, and a fresh set of future events is calculated to reflect this.

For each event in temporal turn, the state of the system is updated using the natural mathematical rules (such as a polymerase position being offset by the product of its speed and the time until the event). Then the event is handled (e.g., polymerase’s speed is set to zero if the event is a collision). The list of events is then updated in light of the previous two steps: e.g., in the collision case, the next collision-time is recalculated to be the time taken for the polymerase immediately prior to the one stalled to reach it; but time-to-repair is simply reduced by the amount of time elapsed prior to the collision event. The events are then re-sorted to find the event that is going to happen next in time, and the process is repeated until the required amount of time has elapsed.

The overall state of the system is a small number of representative genes, and a number of polymerases, each either positioned along one of the genes or available for initiation on any of the genes. There is an initial period to achieve steady state, where polymerases are allowed to initiate on the genes (weighted, to allow different proportions of the gene classes) and progress along the gene body without any damage sites. When equilibrium is achieved in terms of initiation and completion, damage sites are introduced at random locations and for random durations (tunable by the user) which will halt any polymerase that encounters them.

Moving polymerases have a probability of being removed from the gene-body at any moment in time; halted polymerases (at a damage site, or in a queue forming to its 3′ side) can have an increased risk. Also, upon removal polymerases have a probability of being returned to the pool of polymerases available for initiation, or conversely being degraded. A separate parameter is available to similarly determine the fate of polymerases that have completed transcription.

As the damage sites are stochastic, we run the simulation independently one hundred times to achieve an estimate of polymerase density across the gene bodies of a pool of cells.

The dynamics, and the impact each event has, are tunable by the set of parameters given in Table S3. The source code for the simulation is available at https://github.com/FrancisCrickInstitute/babs_uv_polymerase, which also contains a list of all current sites where an interactive version is available.

#### Computational analysis of genome-wide experiments

##### TT-seq (nascent RNA-seq) alignment and quantification

Experiment was performed in biological triplicate. Reads were aligned against the *Homo sapiens* GRCh38 and *Saccharomyces* cerevisiae sacCer3 genome builds using STAR v2.5.2a (Dobin et al., 2013) with Ensembl release 89 transcript annotations. The yeast spike-in was used to account for differences in library size between samples. A yeast gene-level counts matrix was generated using the GenomicAlignments (Lawrence et al., 2013) package’s SummarizeOverlaps function (mode = ”Union,” ignore.strand = FALSE) and passed to DESeq2′s estimateSizeFactors function (Love et al., 2014). An equivalent human count matrix was further analyzed with DESeq2 using the yeast-derived scale factors.

##### Nascent RNA-seq profiles

ngs.plot software (Shen et al., 2014) was used to generate read coverage profiles over the TSS (−5kb:+100kb) and gene-bodies (−5kb:+5kb) of protein coding genes. Yeast-derived scale factors were used to normalize coverage between samples. Genes were stratified by genomic width where appropriate.

##### PolyA mRNA-seq alignment, quantification and differential expression

Experiment was performed in biological triplicate. Reads were Illumina adaptor trimmed using Cutadapt v1.9.1 (Martin, 2011) and aligned against GRCh38 and Ensembl release 89 transcript annotations using STAR v2.5.2a (Dobin et al., 2013) via the transcript quantification software RSEM v1.2.31 (Li and Dewey, 2011). Gene-level counts were rounded to integers and subsequently used for differential expression analysis with DESeq2 (Love et al., 2014). Default size factors were replaced with ones derived from an ERCC spike-in counts matrix using DESeq2′s estimateSizeFactors function (Love et al., 2014). Differential expression results were thresholded for significance based on an FDR ≤ 0.01, a fold-change of ± 2, and a base-mean expression of > = 10. Significant genes were further restricted to a protein-coding set. The top 50 (25 up and 25 down ordered on fold-change) significant genes at the 8h time-point was used to create an expression heatmap from the rlog transformed count data, scaled across samples using a z-score. Genes were stratified into groups based on genomic width: short (< 30kb), medium (> = 30kb: < 100kb) and long (> = 100kb).

##### Alternative splicing event detection

Alternative splicing events were detected in the mRNA-seq data using the Bioconductor package EventPointer (Romero et al., 2016). A splice graph constructed from v89 of the Ensembl human transcriptome and the aligned reads from all BAM files was used to detect and quantify a range of alternative splicing events. Events were quantified and a differential detection analysis was conducted between *K*_*1268*_*R* mutant and WT samples at individual time-points. AFE/ALE and A3SS/A5SS annotations were swapped for genes on the negative strand for consistency.

##### Gene Set Enrichment Analysis (GSEA)

The Broad’s Gene set enrichment analysis (GSEA) software (Subramanian et al., 2005) was used to assess whether gene length shows a significant, concordant difference between expression of the K1268R mutant and WT samples (24h). The GseaPreranked tool (scoring_scheme = classic) was used in conjunction with custom sets of genes created based on width (> 0: < 1, > = 1: < 5, > = 5: < 10, > = 10: < 25, > = 25: < 50, > = 50: < 75, > = 75 kb) and genes pre-ranked based on the Wald statistic from the differential expression analysis.

##### BigWig files

For the purposes of visualization, genome alignment BAM files were merged across biological replicates, sorted and indexed using Picard v2.1.1 (http://broadinstitute.github.io/picard). bigWig files were generated by converting BAM files to bedGraph format using BEDtools’ genomeCoverageBed function (Quinlan and Hall, 2010). Where applicable, yeast scale factors were applied to normalize for differences in library size. bedGraph files were in turn converted to bigWig format using the bedGraphToBigWig function from the KentTools package (Kent et al., 2010).

#### RPB1 loop sequence alignment and phylogenetic study

Orthologous RPB1 protein sequences from common eukaryotic model organisms were downloaded from the Uniprot database and aligned using ClustalW2 (Thompson et al., 1994). The alignment was restricted to the loop region by trimming at residues 1242-1299 of the human protein (RPB1_HUMAN) and visualized using Jalview (Waterhouse et al., 2009).

### Data and Code Availability

Genome-wide data used in this study are available under GEO number GSE143542. The source code for the mathematical modeling of transcription is available at https://github.com/FrancisCrickInstitute/babs_uv_polymerase, which also contains a list of all current sites where an interactive version is available.

## Supplemental Information

Table S1. RPB1 Ubiquitylation Sites Found in Mass Spectrometry Studies, Related to Figure 1 An Excel file listing all ubiquitylation sites detected on RPB1, in two studies (Boeing et al., 2016; Elia et al., 2015).

Table S2. Proteome-wide Ubiquitylation Analysis at Different Time Points after UV Irradiation, Related to Figure 3 An Excel file providing raw data, and fold changes, of all detected ubiquitylation sites in the proteome, in untreated WT cells and at different times after UV irradiation (20 J/m2).

Table S3. Parameters Used for RNAPII Activity Simulation, Related to Figures 3 and 4 A list of all parameter values used to simulate RNAPII activity.

Table S4. Oligonucleotides, Related to STAR Methods
