# Supplementary material for: Regulation of the RNAPII Pool Is Integral to the DNA Damage Response
Source: Cell. 2020 Mar 19;180(6):1245–1261.e21. doi: 10.1016/j.cell.2020.02.009 (PMC7103762; doi:10.1016/j.cell.2020.02.009)
Supplement: Table S4. Oligonucleotides, Related to STAR Methods [file mmc4.pdf]

Table S4. Oligonucleotides. Related to STAR Methods.

REAGENT or RESOURCE	SOURCE	IDENTIFIER
**HDR CRISPR: gRNA sequences**		
K1268R_gRNA_F	CACCGCGATGAGAACAAGATGCAAG	N/A
K1268R_gRNA_R	aaacCTTGCATCTTGTTCTCATCGC	N/A
K1350R_gRNA_F	CACCGATGCGGGTGCTGAGTGAGA	N/A
K1350R_gRNA_R	aaacTCTCACTCAGCACCCGCATC	N/A
ERCC6_ngRNA_1_REV	aaacCACAGCAGAGGTGGACAGCC	N/A
ERCC6_ngRNA_1_FWD	CACCGGCTGTCCACCTCTGCTGTG	N/A
ERCC6_ngRNA_2_FWD	CACCGCACCCACAGAACGAAAGGAG	N/A
ERCC6_ngRNA_2_REV	aaacCTCCTTTCGTTCTGTGGGTGC	N/A
**HDR CRISPR: ssDON sequences**		
K1268R_ssDNA_donor	AATGTTGATTGCATCTTCTTTTCTTTGGTCTCTAACGTGATTCATGCGTTTTATACATGTGACACGCTGACTTCTAGGACCCCCATTATCTCTTGCATCCTGTTCTCATCGGAGTTCATGATGCGAATACGGAGCACCAGCTTCTCTGCATTGTCATCATTA	N/A
K1350R_ssDNA_donor	GATCCACGAAAGGCAGCTAGGCAGCACACACGGGCTCACCGTGAAGATCTCCACAATGTCATTGGACGTGGTGCGTACGGGGTCCACGTCGCêGCTCTGACAGCACCCGCATCAAGCTCACGCCGTCCGTCTCCAGGATCCAC	N/A
**nascent RNA qPCR primers**		
EXT1-gene end F	TCAGGGTAAACAAGGGCAAC	N/A
EXT1-gene end R	CATCCTGGAGGATTGTTCGT	N/A
EXT1-TSS proximal F	CTCAGTTCCAGGCTCAAAGG	N/A
EXT1-TSS proximal R	CACAAGGATTCTCGCTGTGA	N/A

FOS-nascent F	CCGGGGATAGCCTCTCTTAC	N/A
FOS-nascent R	GGGAGCCCCCTACTCATCTA	N/A
LMNB1-gene body F	TGGCCAGATCTTGTGGTTCC	N/A
LMNB1-gene body R	GTGGAGAGCAGCATTTTGGC	N/A
NRIP1-TSS proximal F	CCTGTCCGCCTCTAAGACAC	N/A
NRIP1-TSS proximal R	ATGAAGGTTTCCGTGGATGT	N/A
PUM1-gene end F	AATCACTCGGCAGCCATAAG	N/A
PUM1-gene end R	CTGAAGCATTGAAGGTGGTG	N/A
TMSB10-nascent F	AGTGGGTTTGCAAGGAAGAG	N/A
TMSB10-nascent R	AATCGCCAGCTTCGATAAGG	N/A
ZNF644-TSS proximal F	AGGGGAGGGGTGTAGACAGT	N/A
ZNF644-TSS proximal R	TAGTCACATGAAGCCGAGCA	N/A
**mature RNA qPCR primers**		
ATF3 F	CATCCAGAACAAGCACCTCTG	N/A
ATF3 R	TTCTCGTCGCCTCTTTTTCC	N/A
EGR1 F	CAGCACCTTCAACCCTCAG	N/A
EGR1 R	AGCACCTTCTCGTTGTTCAG	N/A
FOS F	CCGGGGATAGCCTCTCTTAC	N/A
FOS R	GTGGGAATGAAGTTGGCACT	N/A
FOSB F	TGTCTTCGGTGGACTCCTTC	N/A
FOSB R	ACTGCTGTAGCCACTCATGC	N/A
GAPDH F	CAGCCTCAAGATCATCAGCA	N/A
GAPDH R	TGAGTCCTTCCACGATACCA	N/A
**Site-directed mutagenesis primers**		
K1268R F	TGATGAGAACAGGATGCAAGAAG	N/A
K1268R R	CTGTTCATGATGCGAATAC	N/A
K(619, 627, 642)R F	GAGAATGGGGAGCTGATCATGGGCATCCTGTGTA	N/A

	GGAAGTCTCTGGGCACGTCA	
K(619, 627, 642)R R	CACCACCACCCTGGTGTCCCCAGGAGAGATGTGCCTGTAAGGGCCACTGTCTTC	N/A
K(710,719)R F	GAAGTGATTGAAAGGGCACACAACAATGAGCTG	N/A
K(710,719)R R	GATAACGTCCTGCCTGGCCTTCTTAATAGTGTTC	N/A
K(751, 758, 767)R F	CCTGTCTGAATACAACAACTTCAGGTCTATGGTCGTGTCCGGA	N/A
K(751, 758, 767)R R	GATCTCTGAGCAGAGGAGCCAGTCCTGTCTCGGGCATCGTTAAG	N/A
K1278R F	GGTGGTGGACAGGATGGATGATG	N/A
K1278R R	TCTTCCTCTTCTTGCATCTTG	N/A
